# Prevalence of Incidental Findings Suspicious for Transthyretin Cardiac Amyloidosis among Patients Undergoing Bone Scintigraphy: A Systematic Review and a Meta-Analysis

**DOI:** 10.3390/jcm12175698

**Published:** 2023-09-01

**Authors:** Giorgio Treglia, Chiara Martinello, Francesco Dondi, Domenico Albano, Francesco Bertagna, Alessio Rizzo, Roberto C. Delgado Bolton, Gregorio Tersalvi, Barbara Muoio, Martin Riegger, Diego Cecchin

**Affiliations:** 1 Division of Nuclear Medicine, Imaging Institute of Southern Switzerland, Ente Ospedaliero Cantonale, 6501 Bellinzona, Switzerland; 2 Faculty of Biomedical Sciences, Università della Svizzera Italiana, 6900 Lugano, Switzerland; 3 Faculty of Biology and Medicine, University of Lausanne, 1011 Lausanne, Switzerland; 4 Department of Nuclear Medicine and Molecular Imaging, Lausanne University Hospital (CHUV), 1011 Lausanne, Switzerland; 5 School of Medicine, University of Padua, 35128 Padua, Italy; 6 Division of Nuclear Medicine, Università degli Studi di Brescia and ASST Spedali Civili di Brescia, 25123 Brescia, Italy; 7 Department of Nuclear Medicine, Candiolo Cancer Institute, 10060 Turin, Italy; 8 Department of Diagnostic Imaging (Radiology) and Nuclear Medicine, University Hospital San Pedro and Centre for Biomedical Research of La Rioja (CIBIR), 26006 Logroño, Spain; 9 Servicio Cántabro de Salud, 39011 Santander, Spain; 10 Department of Cardiology, Cardiocentro Ticino Institute, Ente Ospedaliero Cantonale, 6900 Lugano, Switzerland; 11 Department of Internal Medicine, Ente Ospedaliero Cantonale, 6850 Mendrisio, Switzerland; 12 Division of Medical Oncology, Oncology Institute of Southern Switzerland, Ente Ospedaliero Cantonale, 6501 Bellinzona, Switzerland; 13 Division of Orthopedics and Traumatology, Department of Surgery, Ente Ospedaliero Cantonale, 6900 Lugano, Switzerland; 14 Nuclear Medicine Unit, Department of Medicine (DIMED), Padova University Hospital, 35128 Padua, Italy

**Keywords:** amyloidosis, transthyretin, bone scan, scintigraphy, cardiac uptake, incidental, meta-analysis, nuclear medicine

## Abstract

Background: The myocardial uptake of bone-seeking tracers suspicious for transthyretin cardiac amyloidosis (ATTR-CA) can be incidentally detected in patients undergoing bone scintigraphy for noncardiac reasons. We conducted a systematic review and meta-analysis to assess the prevalence of these scintigraphic findings. Methods: A comprehensive literature search was performed using two bibliographic databases (PubMed/MEDLINE and Cochrane Library), searching for articles related to the review question. Eligible articles were selected, and relevant data were extracted by two authors. The pooled prevalence of incidental findings suspicious for ATTR-CA among patients undergoing bone scintigraphy was calculated on a per-patient-based analysis using a random-effects model. The pooled measure was provided with 95% confidence interval (95% CI) values. Results: Among 219 records, 11 articles were selected for the systematic review and 10 for the meta-analysis. The pooled prevalence of incidental findings suspicious for ATTR-CA was 1.1% (95% CI: 0.7–1.4%) with heterogeneity due to the characteristics of the included studies, patients, and index tests. These findings are more prevalent in older men. Conclusions: The prevalence of incidental findings of ATTR-CA among patients undergoing bone scintigraphy is low but not negligible. Nuclear medicine physicians should suggest, in the scintigraphic report, further clinical investigations when these findings are detected. Prospective studies are warranted.

## 1. Introduction

Cardiac amyloidosis (CA) is a serious and progressive infiltrative disease caused by the deposition of amyloid fibrils in the myocardium. In most cases, it leads to heart failure, reduced quality of life, and death [1,2,3,4,5]. There are two main subtypes of CA, transthyretin amyloidosis (ATTR-CA) and immunoglobulin light chain amyloidosis (AL-CA), each associated with specific underlying protein abnormalities. Due to its often nonspecific and subtle initial symptoms, CA can be challenging to diagnose, leading to delayed recognition and treatment. Thanks to advances in imaging techniques and the possibility of achieving a noninvasive diagnosis, CA is more frequently encountered than it used to be [1,2,3,4,5]. The epidemiological distribution of CA has changed over recent years, particularly due to the advances in diagnostic methods and therapeutic options in the field of ATTR-CA [6]. In this regard, the possibility of employing bone scintigraphy (also with actual novel 3D solid state detectors) to diagnose ATTR-CA without a biopsy has revealed the real prevalence of the disease. Early detection and appropriate management of ATTR-CA are crucial to improving patient outcomes, even more so considering recent promising therapies that interrupt amyloid deposition or possibly remove amyloid fibrils [6].

The most promising imaging modality for diagnosis of ATTR-CA is bone scintigraphy with technetium 99m (^99m^Tc)-radiolabeled bone-seeking tracers, including pyrophosphate (PYP), 3,3-diphosphono-1,2-propanodicarboxylic acid (DPD), and hydroxymethylene diphosphonate (HMDP) [7,8,9]. Bone scintigraphy complements cardiac structural and functional characterization through echocardiography and cardiac magnetic resonance in the diagnosis of ATTR-CA. The rationale for using bone scintigraphy to differentiate between ATTR-CA and AL-CA is that ATTR-CA has avidity for bone-seeking radiotracers, whereas AL-CA has minimal or no avidity for these tracers. Therefore, bone scintigraphy can diagnose ATTR-CA when plasma cell dyscrasia is excluded [7,8,9]. Evidence-based data demonstrated that, in patients with suspicious cardiac amyloidosis, bone scintigraphy has high diagnostic accuracy for diagnosis of ATTR-CA [10].

Beyond the use of bone scintigraphy for diagnosis of ATTR-CA, myocardial uptake of bone-seeking tracers, suspicious for ATTR-CA, can be incidentally detected in patients undergoing bone scintigraphy for noncardiac reasons (including oncological, orthopedical, and rheumatological indications). The knowledge of the prevalence of incidental findings suspicious for ATTR-CA in bone scintigraphy may unveil the real prevalence of this disease [6]. Therefore, we performed an updated systematic review and meta-analysis to evaluate the prevalence of incidental findings suspicious for ATTR-CA among patients undergoing bone scintigraphy for noncardiac diseases.

## 2. Materials and Methods

### 2.1. Protocol

This evidence-based article was created according to a predefined protocol [11]; the protocol was not registered in PROSPERO as the protocol registration is not mandatory. The article was written according to the latest version of “Preferred Reporting Items for a Systematic Review and Meta-Analysis (PRISMA) statement” [12]. The complete PRISMA checklist is available in the Supplementary Materials (Table S1).

The review question was the assessment of the prevalence of incidental findings suspicious for ATTR-CA among patients undergoing bone scintigraphy for noncardiac diseases.

### 2.2. Literature Search

A comprehensive literature search was performed independently by two authors using two electronic bibliographic databases (PubMed/MEDLINE and Cochrane Library), searching for studies evaluating the prevalence of incidental findings suspicious for ATTR-CA among patients undergoing bone scintigraphy. The bibliographic databases were searched until 31 March 2023. A search algorithm based on a combination of text words related to the review question was used: (A) “bone” OR “skeletal” OR “technetium” OR “pyrophosphate” OR “PYP” OR “DPD” OR “HMDP” OR “HDP” OR “MDP” OR “diphosphonat*” AND (B) “cardiac” OR “myocard*” OR “heart” AND (C) “amyloid*” AND (D) “inciden*” OR “prevalence”. Date limits or language restrictions were not applied to the search of electronic databases. Furthermore, to achieve a more comprehensive search, the references of retrieved studies were also searched for potential additional eligible articles. In other words, the reference lists of retrieved full-text articles were screened in the search for articles that could be eligible for the systematic review, taking into account their titles.

### 2.3. Eligibility Criteria

Clinical studies reporting information on the prevalence of incidental findings suspicious for CA among patients undergoing bone scintigraphy for non-cardiological indications were eligible for inclusion in the systematic review and meta-analysis. Exclusion criteria were review articles/letters/comments/editorials in the topic of interest; case reports/small case series in the topic of interest; and articles outside the field of interest. If articles with patient data that possibly overlapped with another study were retrieved, all the selected articles were included in the systematic review (qualitative synthesis), whereas only the article with the most complete information was included in the meta-analysis (quantitative synthesis).

### 2.4. Study Selection

Titles and abstracts of the records retrieved using the search strategy were independently screened by two authors based on the predefined inclusion and exclusion criteria mentioned above. The full texts of selected original articles were independently screened to assess for their final inclusion in the systematic review and meta-analysis. For all the screened records using the bibliographic databases, the reviewers provided a final decision on inclusion or exclusion in the review, specifying the reason. Disagreements between the authors were solved through the involvement of a third author.

### 2.5. Data Collection and Extraction

The data collection and extraction were independently carried out by two authors to minimize possible bias. Full texts, tables, and/or figures from the selected reports were analyzed for data extraction. In selected cases, reviewers contacted corresponding authors by e-mail to obtain missing data. Data extracted through piloted forms included general study information (authors, year of publication, country, study design, and funding sources); patient characteristics (sample size, age, sex ratio, and clinical indications for undergoing bone scintigraphy); index text characteristics (type of bone-seeking tracer used during bone scintigraphy, date of examination, and bone scintigraphy protocol); and main outcome (number and prevalence of incidental findings suspicious for ATTR-CA at bone scintigraphy). Any discrepancy between the authors about data extraction was solved by a third reviewer.

### 2.6. Quality Assessment

QUADAS-2 was used as tool for assessing risk of bias in individual studies and concerns regarding applicability [13]. Quality assessment was independently carried out by two authors and any discrepancy was solved by a third author.

### 2.7. Statistical Analysis

The prevalence of incidental findings suspicious for ATTR-CA among patients undergoing bone scintigraphy was calculated, taking into account data extracted from each study. Pooled prevalence was calculated on a per patient basis using a random-effects model. The pooled measure was provided with 95% confidence interval values (95% CI) and displayed using forest plots. Statistical heterogeneity was assessed using the I-square or inconsistency index (I^2^), with significant heterogeneity for values > 50% [11]. Subgroup analyses were planned in case of significant heterogeneity. Egger’s test was carried out to calculate the publication bias. OpenMeta[Analyst]^®^ software (Rockville, MD, USA) was used for the statistical analyses.

## 3. Results

### 3.1. Literature Search and Selection of Studies

**Figure 1:**
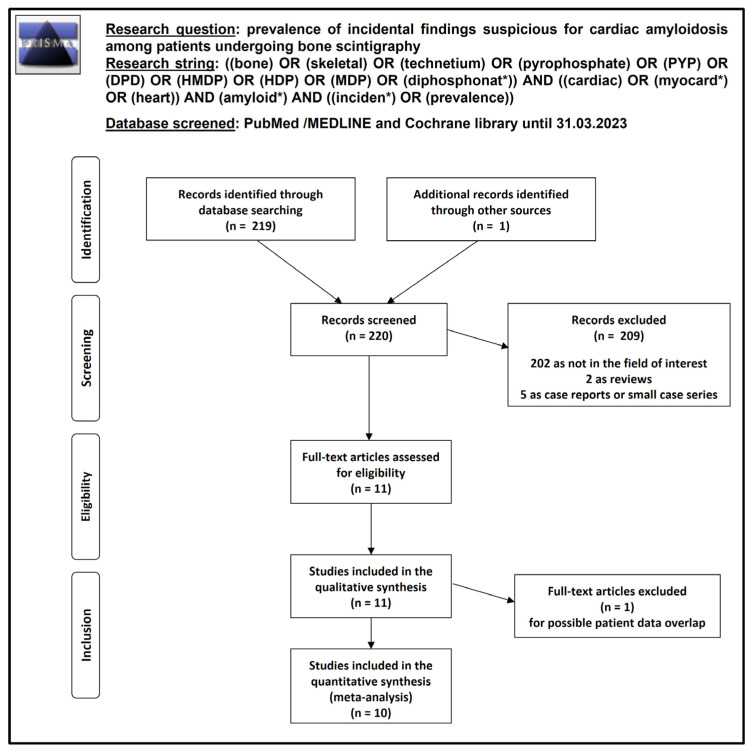
Summary of selection process of eligible articles for the systematic review and meta-analysis. *: it is a truncation.

Overall, 219 records were identified and screened through the comprehensive literature search: 209 records were excluded using the predefined eligibility criteria (202 as not in the field of interest, 2 as reviews, and 5 as case reports or small case series), and 10 were selected [14,15,16,17,18,19,20,21,22,23]. One record was added after the references of potentially eligible studies were screened [24]. One study was included in the systematic review but excluded from the meta-analysis due to a possible patient data overlap with another study by the same group of researchers [16]. Overall, after full-text assessment, 11 studies were judged as eligible for inclusion in the systematic review (qualitative synthesis) [14,15,16,17,18,19,20,21,22,23,24] and 10 were included in the meta-analysis (quantitative synthesis) [14,15,17,18,19,20,21,22,23,24]. Figure 1 summarizes the study selection process. The list of included and excluded studies (with explanation) is available in the Supplementary Materials (Table S2).

### 3.2. Study Characteristics

The characteristics of the 11 studies eligible for the systematic review (qualitative analysis), which included more than 63,000 patients, are presented in Table 1, Table 2 and Table 3.

Regarding the general study information (Table 1), these studies were published from 1995 to 2023. Several countries were represented, but mainly European countries. All the included studies were retrospective analyses. All except two were monocentric studies. In most of the studies, no funding source was declared.

Regarding the characteristics of the included patients (Table 2), the mean age ranged from 61 to 80 years, the male percentage ranged from 37% to 82%, and the clinical indications for performing bone scintigraphy were in all cases the detection of bone metastases and benign skeletal conditions.

Regarding bone scintigraphy (Table 3), different bone-seeking radiopharmaceuticals were used, but the most frequent were ^99m^Tc-DPD and ^99m^Tc-HMDP. The injected activity and the imaging protocol largely varied among the included studies. However, in all the included studies planar whole-body scintigraphic images in the anterior–posterior view were acquired. The interpretation of scintigraphic images was conducted through a visual analysis in all the included studies and a grading score was used to compare myocardial tracer uptake with bone tracer uptake (grade 0: no myocardial tracer uptake; grade 1: mild myocardial tracer uptake, inferior to bone uptake; grade 2: moderate myocardial tracer uptake, equal to bone uptake; grade 3: strong myocardial tracer uptake, higher than bone uptake). In a few studies, an additional semiquantitative analysis (using the heart to whole-body uptake ratio or the heart to contralateral uptake ratio) was performed. Diffuse myocardial tracer uptake equal to or higher than bone tracer uptake was usually considered a scintigraphic finding suspicious for ATTR-CA in the included studies. There were usually two or three readers of the scintigraphic images (when declared), reaching a consensus in case of discordant findings.

### 3.3. Quality (Risk of Bias) Assessment

**Figure 2:**
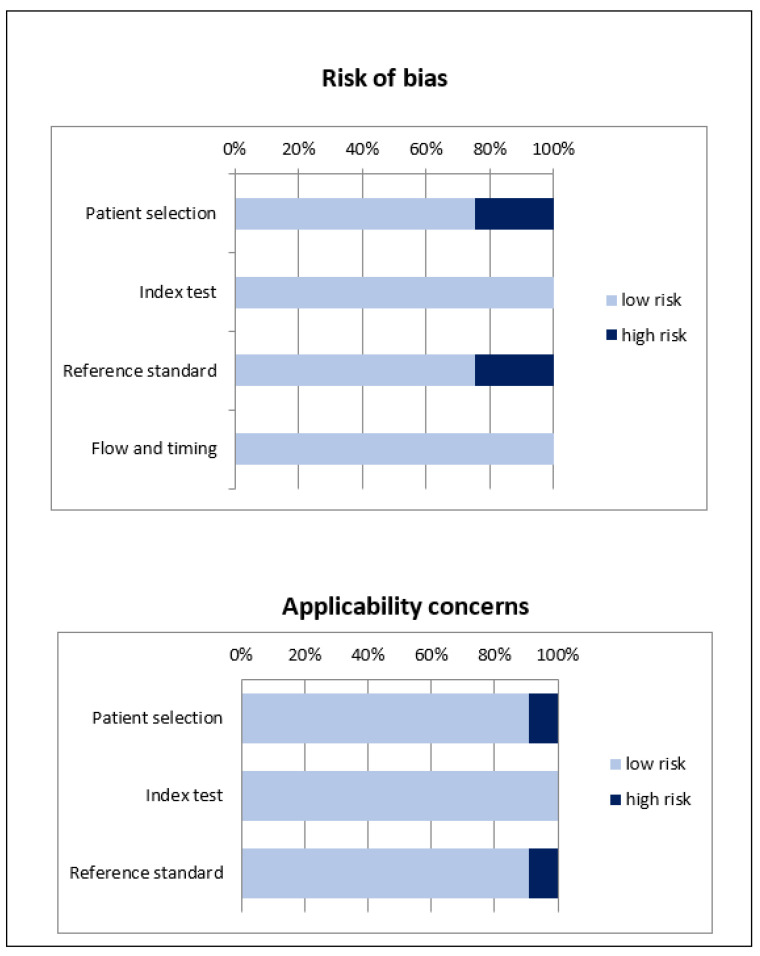
Overall quality assessment using QUADAS-2 tool. Studies included in the systematic review are classified as at low risk or high risk of bias or applicability concerns for different domains.

The overall evaluation of risk of bias and concerns regarding applicability for studies included in the systematic review according to QUADAS-2 is presented in Figure 2.

### 3.4. Systematic Review (Qualitative Synthesis of Results)

The prevalence and characteristics of patients with incidental findings suspicious for ATTR-CA at bone scintigraphy are illustrated in Table 4. The overall prevalence of incidental myocardial uptake suspicious for ATTR-CA varied between 0.1% and 3.4%, but it increased progressively with age, showing higher values in patients with age > 80 years [14,15,18,20,22,23]. Patients with myocardial tracer uptake were significantly older (mean age from 78 to 86 years) than those with no myocardial tracer uptake [14,15,17,19,22]. Regarding gender, males represented the majority of patients with incidental myocardial uptake of bone-seeking tracers suspicious for ATTR-CA (from 62% to 90%) [14,15,16,17,18,19,20,21,22,23,24]. Beyond age and male gender, some studies identified other variables as independent predictors for myocardial tracer uptake suspicious for ATTR-CA [14,15]. Through a multivariate logistic regression analysis using an odds ratio (OR) as an effect measure, Navarro-Saez et al. identified the following variables as independent predictors for myocardial tracer uptake suspicious for ATTR-CA at a bone scan: age (OR: 1.2), male sex (OR: 2.1), arterial hypertension (OR: 3.1), heart failure (OR: 5.4), atrial fibrillation (OR: 2.6), atrioventricular block (OR: 2.5), aortic valve stenosis (OR: 2.2), and carpal tunnel syndrome, either unilateral (OR: 4.4) or bilateral (OR: 146.2) [14].

Few studies correlated the prevalence of incidental findings suspicious for ATTR-CA with the different indications for undergoing bone scans with conflicting results (Table 5).

Most of the patients with scintigraphic findings suspicious for ATTR-CA were asymptomatic, and they usually showed a degree of involvement on echocardiography [18,20]. Tissue biopsy, including Congo red staining and immunohistochemical analysis for confirmation of ATTR-CA in the scintigraphic findings, was only available for a few patients.

The incidental detection of the myocardial uptake of bone-seeking tracers may also have a predictive and a prognostic value [15,17,19,22]. Significant myocardial uptake (grade ≥ 2) was associated with adverse outcomes and an increased risk of hospitalization due to heart failure, resulting in an independent predictor of overall and cardiovascular mortality [15,17,19,22].

### 3.5. Meta-Analysis (Quantitative Synthesis of Results)

**Figure 3:**
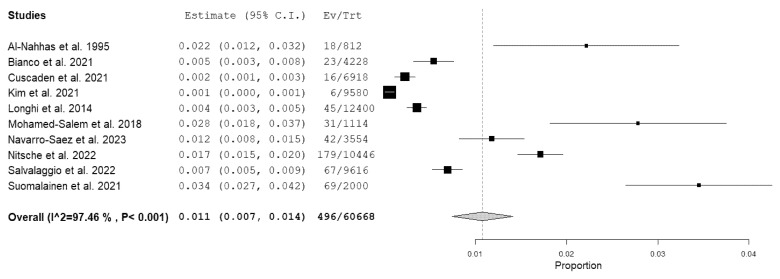
Forest plot of the prevalence of incidental findings suspicious for transthyretin cardiac amyloidosis among patients undergoing bone scintigraphy for noncardiac reasons. [14,15,17,18,19,20,21,22,23,24].

Ten studies were selected for the patient-based meta-analysis on the prevalence of incidental findings suspicious for ATTR-CA at bone scintigraphy. The pooled prevalence was 1.1% (95% CI: 0.7–1.4%). A forest plot is shown in Figure 3.

A significant statistical heterogeneity among the included studies was found (I^2^ = 97%).

The Egger’s test did not demonstrate a significant publication bias (*p* = 0.2).

Subgroup analyses did not show a significant difference in the pooled prevalence values taking into account the different bone-seeking tracers used: the pooled prevalence values using ^99m^Tc-HMDP and ^99m^Tc-DPD were 1.3% (95% CI: 0.4–2.3) and 0.8% (95% CI: 0.3–1.3%), respectively. Conversely, a higher pooled prevalence was observed when comparing European countries (1.5%; 95% CI: 1–1.9%) and non-European countries (0.1%; 95% CI: 0–0.3%). Subgroup analyses did not show a significant difference in the pooled prevalence values when taking into account different publication years: for articles published after 2020 and before 2020, the pooled values were 1% (95% CI: 0.6–1.4%) and 1.7% (95% CI: 0–3.5%), respectively.

## 4. Discussion

The present systematic review and meta-analysis demonstrate that incidental findings of ATTR-CA at bone scintigraphy are not rare (about 1% of all patients undergoing bone scans for noncardiac reasons).

ATTR-CA is characterized by a long latency period between the onset of symptoms and the definitive diagnosis [1,2,3]. Therefore, incidental findings of ATTR-CA at bone scintigraphy should be considered as a crucial opportunity to diagnose this condition early in the disease course. Nuclear medicine physicians should suggest, in the scintigraphic report, further clinical evaluation when suspicious findings for ATTR-CA are detected during bone scans to allow a complete diagnostic workup of these patients and to avoid the loss of ATTR-CA patients at follow-up. As the only currently approved therapy for ATTR-CA is the transthyretin stabilizer Tafamidis, which works by slowing down the deposition of fibrils, diagnosis of ATTR-CA in a preclinical stage could allow early treatment, potentially improving the prognosis [1,2,3].

Taking into account data from the included studies, incidental findings suspicious for ATTR-CA at bone scintigraphy increased in frequency with age and were more common in male than in female patients [14,15,16,17,18,19,20,21,22,23,24]. These findings are not surprising but are consistent with the age and gender distributions of patients who present clinically with heart failure due to ATTR-CA [1,2,3].

Notably, beyond older age and male sex, multiple conditions including arterial hypertension, aortic stenosis, atrioventricular block, atrial fibrillation, and carpal tunnel syndrome were predictors of myocardial uptake of bone-seeking tracers [14,15]. This finding seems not surprising as these conditions are already known to be related to ATTR-CA [6]. In patients undergoing bone scintigraphy, the documentation of multiple risk factors could prompt a referral to an expert unit for extended studies to rule out the presence of ATTR-CA.

Interestingly, the incidental detection of myocardial uptake of bone-seeking tracers may also have a predictive and a prognostic value [15,17,19,22]. Significant myocardial uptake (grade ≥ 2) was associated with adverse outcomes and an increased risk of hospitalization due to heart failure, resulting in an independent predictor of overall and cardiovascular mortality [15,17,19,22]; these findings further highlight the importance of a timely and appropriate diagnosis of ATTR-CA by using bone scintigraphy.

Our evidence-based manuscript has some limitations, most of them related to the characteristics and study design of the included studies. First of all, all the included studies are retrospective and most of them are monocentric. Second, the final diagnosis of ATTR-CA was not available for all suspicious patients; therefore, an underestimation/overestimation of the true prevalence of ATTR-CA cannot be excluded due to possible false negative (i.e., some transthyretin gene mutations) or false positive results (i.e., myocardial scar due to previous myocardial infarction or some cases of AL-CA) of bone scintigraphy, which should be taken into account in an unselected population in particular. The specificity of diagnosing ATTR-CA using bone scintigraphy is markedly improved when the presence of a monoclonal gammopathy is excluded [4,5]. However, the articles included in this meta-analysis did not involve performing bone scintigraphy for diagnosis of ATTR-CA and the outcome measures used were not sensitivity or specificity. The included articles evaluated the prevalence of incidental findings suspicious for ATTR-CA in an unselected population with unknown cardiac amyloidosis; therefore, laboratory testing for monoclonal gammopathy was not performed before bone scans were performed. A visual grade ≥ 2 was usually used as a surrogate marker for the presence of ATTR-CA, but this is justified by evidence-based data reporting that the pooled sensitivity, specificity, and positive predictive value of bone scintigraphy for ATTR-CA diagnosis using visual grade ≥ 2 as a positive finding were 92%, 95%, and 96%, respectively [10]. Another limitation could be that the selected studies included only patients who underwent bone scintigraphy for several clinical indications, and this sample may be not representative of the general population; however, individuals referred for bone scintigraphy to assess cardiac diseases were excluded from the meta-analysis, and hence it is reasonable to consider the included patients as representative of the general population of equivalent age in regards to the presence of cardiac disease. Lastly, heterogeneity among the included studies, mainly due to different characteristics of patients and index tests (technology aspects), is a limitation of our meta-analysis. To explore the heterogeneity of our meta-analysis, we planned subgroup analyses taking into account the different bone-seeking tracers and different countries (European versus non-European countries). Interestingly, we did not find a significant difference among the different bone-seeking tracers (e.g., DPD versus HMDP), but only among the different countries, likely due to the differing epidemiology of the disease. For instance, taking into account the countries of the articles included in this systematic review, the prevalence of ATTR-CA in European countries is expected to be higher than that in Asia–Oceania, even if regional and genetic differences among different countries should be considered [25,26,27,28].

An advantage of our meta-analysis is the absence of a significant publication bias.

Overall, the findings reported in our evidence-based article support the hypothesis that amyloid deposition in ATTR-CA is a continuous process and that the myocardial uptake of bone-seeking tracers precedes clinical manifestations of heart failure, allowing the early diagnosis of ATTR-CA even before the appearance of other echocardiographic and electrocardiographic abnormalities or overt clinical manifestations.

Beyond prospective studies on the prevalence of incidental detection of ATTR-CA in patients undergoing bone scintigraphy, future perspectives are represented by the use of artificial intelligence and machine learning algorithms or new semiquantitative methods to accurately detect and classify different grades of myocardial tracer uptake on bone scintigraphy, allowing for better detection of suspicious ATTR-CA cases [29,30,31,32,33,34,35]. Furthermore, more studies evaluating the possible correlations among the different indications for bone scintigraphy and incidental detection of ATTR-CA should be carried out. Through bone scintigraphy, it could also be theoretically possible to evaluate the possible correlation between diseases with impaired bone metabolism and ATTR-CA [36].

## 5. Conclusions

The prevalence of incidental findings of ATTR-CA among patients undergoing bone scintigraphy is low but not negligible, and it increases in older men. Nuclear medicine physicians should suggest, in the scintigraphic report, further clinical investigations when findings suspicious for ATTR-CA are detected. Prospective studies are needed to support the findings of this updated systematic review and meta-analysis.

## Figures and Tables

**Table 1:** General study information.

Authors [Ref.]	Year	Country	Study Design	Funding Sources
Al-Nahhas et al. [24]	1995	United Kingdom	Retrospective monocentric	None declared
Bianco et al. [18]	2021	Italy	Retrospective monocentric	None declared
Cuscaden et al. [20]	2021	Australia	Retrospective monocentric	None declared
Halme et al. [16]	2022	Finland	Retrospective multicentric	None declared
Kim et al. [21]	2019	Korea	Retrospective monocentric	None declared
Longhi et al. [23]	2014	Italy	Retrospective monocentric	None declared
Mohamed-Salem et al. [22]	2018	Spain	Retrospective monocentric	None declared
Navarro-Saez et al. [14]	2023	Spain	Retrospective monocentric	None declared
Nitsche et al. [15]	2022	Austria	Retrospective monocentric	Pfizer
Salvalaggio et al. [17]	2022	Italy	Retrospective monocentric	ALNYLAM Pharmaceuticals Inc.
Suomalainen et al. [19]	2021	Finland	Retrospective multicentric	None declared

**Table 2:** Patient characteristics.

Authors [Ref.]	No. of Patients	Mean Age (Years)	Male %
Al-Nahhas et al. [24]	812	69	69%
Bianco et al. [18]	4228	NR	NR
Cuscaden et al. [20]	6918	NR	53%
Halme et al. [16]	1334 (1319 *)	77	82%
Kim et al. [21]	9580	NR	NR
Longhi et al. [23]	12,400	74	37%
Mohamed-Salem et al. [22]	1114	80	65%
Navarro-Saez et al. [14]	3629 (3554 *)	NR	NR
Nitsche et al. [15]	11,527 (10,446 *)	61	37%
Salvalaggio et al. [17]	9616	79	75%
Suomalainen et al. [19]	2000	78	69%

Legend: * = excluding patients undergoing bone scintigraphy for cardiac conditions. NR = not reported.

**Table 3:** Index text characteristics.

Authors [Ref.]	Tracers Used	Date of Bone Scintigraphy	Injected Activity	Time from Tracer Injection to Image Acquisition	Image Acquisition	Image Interpretation	No. of Readers
Al-Nahhas et al. [24]	^99m^Tc-HDP/^99m^Tc-MDP	1991–1993	NR	NR	Planar WB images in A-P view	Visual (grading of myocardial uptake compared with bone uptake)	3
Bianco et al. [18]	^99m^Tc-HMDP/^99m^Tc-DPD	2015–2020	10 MBq/kg	3 h	Planar WB images in A-P view	Visual (grading of myocardial uptake compared with bone uptake) and semiquantitative (H/CL)	NR
Cuscaden et al. [20]	^99m^Tc-HMDP/^99m^Tc-MDP	2005–2018	NR	NR	Planar WB images in A-P view ± SPET	Visual (grading of myocardial uptake compared with bone uptake) and semiquantitative (H/WB)	2
Halme et al. [16]	^99m^Tc-HMDP	2012–2021	500–700 MBq	3 h	Planar WB images in A-P view	Visual (grading of myocardial uptake compared with bone uptake)	3
Kim et al. [21]	^99m^Tc-DPD	2014–2017	740 MBq	5 min and 3 h	Planar WB images in A-P view	Visual (grading of myocardial uptake compared with bone uptake)	NR
Longhi et al. [23]	^99m^Tc-DPD	2008–2013	740 MBq	5 min and 3 h	Planar WB images in A-P view	Visual (grading of myocardial uptake compared with bone uptake)	NR
Mohamed-Salem et al. [22]	^99m^Tc-HMDP/^99m^Tc-HDP/^99m^Tc-DPD	2010–2016	740 MBq	2–3 h	Planar WB images in A-P view	Visual (grading of myocardial uptake compared with bone uptake)	2
Navarro-Saez et al. [14]	^99m^Tc-DPD	2017–2020	925 MBq	2–3 h	Planar WB images in A-P view	Visual (grading of myocardial uptake compared with bone uptake)	2
Nitsche et al. [15]	^99m^Tc-DPD	2010–2020	700 MBq	3 h	Planar WB images in A-P view ± SPET	Visual (grading of myocardial uptake compared with bone uptake)	2–3
Salvalaggio et al. [17]	^99m^Tc-HMDP/^99m^Tc-DPD	2009–2020	600–800 MBq	2.5–4 h	Planar WB images in A-P view	Visual (grading of myocardial uptake compared with bone uptake)	2
Suomalainen et al. [19]	^99m^Tc-HMDP	2002–2018	NR	3 h	Planar WB images in A-P view ± SPET	Visual (grading of myocardial uptake compared with bone uptake) and semiquantitative (H/CL)	3

Legend: ^99m^Tc = Technetium-99m; A-P = anterior–posterior; DPD = 3,3-diphosphono-1,2-propanodicarboxylic acid; H/CL = heart to contralateral uptake ratio; H/WB = heart to whole-body uptake ratio; HDP = hydroxyethylene diphosphonate; HMDP = hydroxymethylene diphosphonate; MBq = MegaBecquerel; MDP = methylene diphosphonate; NR = not reported; SPECT = single photon emission tomography; WB = whole body.

**Table 4:** Main outcome: prevalence and characteristics of patients with incidental findings suspicious for ATTR-CA at bone scintigraphy.

Authors [Ref.]	No. of Patients with Findings Suspicious for ATTR-CA **	Mean Age (Years)	Male %	Overall Prevalence of Incidental Findings Suspicious for ATTR-CA (%) ***	Subgroup Analysis on ^99m^Tc-HMDP ***	Subgroup Analysis on ^99m^Tc-DPD ***	Suspicious ATTR-CA Confirmed by Pathology
Al-Nahhas et al. [24]	18	80	94%	18/812 (2.2%)	-	-	NR
Bianco et al. [18]	23	83	78%	23/4228 (0.5%)	19/3505 (0.5%)	4/723 (0.6%)	NR
Cuscaden et al. [20]	16	84	94%	16/6918 (0.2%)	15/3472 (0.4%)	-	NR
Halme et al. [16] *	36	NR	NR	36/1319 (2.7%)	36/1319 (2.7%)	-	NR
Kim et al. [21]	6	81	83%	6/9580 (0.1%)	-	6/9580 (0.1%)	1/1
Longhi et al. [23]	45	81	62%	45/12,400 (0.4%)	-	45/12,400 (0.4%)	5/5
Mohamed-Salem et al. [22]	31	85	90%	31/1114 (2.8%)	NC	NC	NR
Navarro-Saez et al. [14]	42	86	71%	42/3554 (1.2%)	-	42/3554 (1.2%)	NR
Nitsche et al. [15]	179	80	74%	179/10,446 (1.7%)	-	179/10,446 (1.7%)	22/28
Salvalaggio et al. [17]	67	78	75%	67/9616 (0.7%)	NC	NC	NR
Suomalainen et al. [19]	69	81	77%	69/2000 (3.4%)	69/2000 (3.4%)	-	NR

Legend: * = partial data overlap with the study of Suomalainen et al. [19]; ** = patients with uptake grades 2 or 3; *** = excluding patients undergoing bone scintigraphy for cardiac conditions; ATTR-CA = transthyretin cardiac amyloidosis; NC = not calculable; NR = not reported.

**Table 5:** Prevalence of incidental findings suspicious for ATTR-CA and different indications of bone scintigraphy.

Authors [Ref.]	No. of Patients with Findings Suspicious for ATTR-CA	Subgroup Analysis for Prostate Cancer	Subgroup Analysis for Other Tumors	Subgroup Analysis for Other Indications
Al-Nahhas et al. [24]	18	14/322 (4.4%)	4/427 (0.9%)	1/216 (0.5%)
Bianco et al. [18]	23	NC	NC	NC
Cuscaden et al. [20]	16	NC	NC	NC
Halme et al. [16] *	36	31/1013 (3.1%)	5/240 (2.1%)	0/55 (0%)
Kim et al. [21]	6	NC	NC	NC
Longhi et al. [23]	45	NC	NC	NC
Mohamed-Salem et al. [22]	31	NC	NC	NC
Navarro-Saez et al. [14]	42	NC	NC	NC
Nitsche et al. [15]	179	NC	NC	NC
Salvalaggio et al. [17]	67	NC	NC	NC
Suomalainen et al. [19]	69	49/1426 (3.4%)	16/384 (4.2%)	4/190 (2.1%)

Legend: * = partial data overlap with the study of Suomalainen et al. [19]; ATTR-CA = transthyretin cardiac amyloidosis; NC = not calculable.

## Data Availability

Data supporting the reported results can be found using the public PubMed/MEDLINE and Cochrane Library databases.

## Supplementary Materials

The following supporting information can be downloaded at https://www.mdpi.com/article/10.3390/jcm12175698/s1: Table S1: PRISMA checklist; Table S2: list of screened records.

## References

[1] Garcia-Pavia P., Rapezzi C., Adler Y., Arad M., Basso C., Brucato A., Burazor I., Caforio A.L.P., Damy T., Eriksson U. (2021). Diagnosis and treatment of cardiac amyloidosis: A position statement of the ESC Working Group on Myocardial and Pericardial Diseases. Eur. Heart J..

[2] Rubin J., Maurer M.S. (2020). Cardiac Amyloidosis: Overlooked, Underappreciated, and Treatable. Annu. Rev. Med..

[3] Bajwa F., O’Connor R., Ananthasubramaniam K. (2022). Epidemiology and clinical manifestations of cardiac amyloidosis. Heart Fail. Rev..

[4] Schwotzer R., Flammer A.J., Gerull S., Pabst T., Arosio P., Averaimo M., Bacher U., Bode P., Cavalli A., Condoluci A. (2020). Expert recommendation from the Swiss Amyloidosis Network (SAN) for systemic AL-amyloidosis. Swiss Med. Wkly..

[5] Condoluci A., Théaudin M., Schwotzer R., Pazhenkottil A.P., Arosio P., Averaimo M., Bacher U., Bode P., Cavalli A., Dirnhofer S. (2021). Management of transthyretin amyloidosis. Swiss Med. Wkly..

[6] Rossi M., Varrà G.G., Porcari A., Saro R., Pagura L., Lalario A., Dore F., Bussani R., Sinagra G., Merlo M. (2022). Re-Definition of the Epidemiology of Cardiac Amyloidosis. Biomedicines.

[7] Khor Y.M., Cuddy S.A.M., Singh V., Falk R.H., Di Carli M.F., Dorbala S. (2023). ^99m^Tc Bone-Avid Tracer Cardiac Scintigraphy: Role in Noninvasive Diagnosis of Transthyretin Cardiac Amyloidosis. Radiology.

[8] Dorbala S., Ando Y., Bokhari S., Dispenzieri A., Falk R.H., Ferrari V.A., Fontana M., Gheysens O., Gillmore J.D., Glaudemans A.W.J.M. (2021). ASNC/AHA/ASE/EANM/HFSA/ISA/SCMR/SNMMI Expert Consensus Recommendations for Multimodality Imaging in Cardiac Amyloidosis: Part 1 of 2-Evidence Base and Standardized Methods of Imaging. Circ. Cardiovasc. Imaging.

[9] Dorbala S., Ando Y., Bokhari S., Dispenzieri A., Falk R.H., Ferrari V.A., Fontana M., Gheysens O., Gillmore J.D., Glaudemans A.W.J.M. (2021). ASNC/AHA/ASE/EANM/HFSA/ISA/SCMR/SNMMI Expert Consensus Recommendations for Multimodality Imaging in Cardiac Amyloidosis: Part 2 of 2-Diagnostic Criteria and Appropriate Utilization. Circ. Cardiovasc. Imaging.

[10] Treglia G., Glaudemans A.W.J.M., Bertagna F., Hazenberg B.P.C., Erba P.A., Giubbini R., Ceriani L., Prior J.O., Giovanella L., Slart R.H.J.A. (2018). Diagnostic accuracy of bone scintigraphy in the assessment of cardiac transthyretin-related amyloidosis: A bivariate meta-analysis. Eur. J. Nucl. Med. Mol. Imaging.

[11] Sadeghi R., Treglia G. (2017). Systematic reviews and meta-analyses of diagnostic studies: A practical guideline. Clin. Transl. Imaging.

[12] Page M.J., McKenzie J.E., Bossuyt P.M., Boutron I., Hoffmann T.C., Mulrow C.D., Shamseer L., Tetzlaff J.M., Akl E.A., Brennan S.E. (2021). The PRISMA 2020 statement: An updated guideline for reporting systematic reviews. J. Clin. Epidemiol..

[13] Whiting P.F., Rutjes A.W., Westwood M.E., Mallett S., Deeks J.J., Reitsma J.B., Leeflang M.M., Sterne J.A., Bossuyt P.M., QUADAS-2 Group (2011). QUADAS-2: A revised tool for the quality assessment of diagnostic accuracy studies. Ann. Intern. Med..

[14] Navarro-Saez M.D.C., Feijoo-Massó C., Bravo Ferrer Z.D.C., Oliva Morera J.C., Balado González A.M., Palau-Domínguez A., Guillamon Toran L., Comet Monte R., Fernández-Codina A. (2023). Trends in diagnosis of cardiac transthyretin amyloidosis: 3-year analysis of scintigraphic studies: Prevalence of myocardial uptake and its predictor factors. Int. J. Cardiovasc. Imaging.

[15] Nitsche C., Mascherbauer K., Calabretta R., Koschutnik M., Dona C., Dannenberg V., Hofer F., Halavina K., Kammerlander A.A., Traub-Weidinger T. (2022). Prevalence and Outcomes of Cardiac Amyloidosis in All-Comer Referrals for Bone Scintigraphy. J. Nucl. Med..

[16] Halme H.L., Ihalainen T., Suomalainen O., Loimaala A., Mätzke S., Uusitalo V., Sipilä O., Hippeläinen E. (2022). Convolutional neural networks for detection of transthyretin amyloidosis in 2D scintigraphy images. EJNMMI Res..

[17] Salvalaggio A., Cipriani A., Righetto S., Artioli P., Sinigiani G., De Michieli L., Cason M., Pilichou K., Cecchin D., Briani C. (2022). Incidental cardiac uptake of ^99m^Tc-diphosphonates is predictive of poor outcome: Data from 9616 bone scintigraphies. J. Nucl. Cardiol..

[18] Bianco M., Parente A., Biolè C., Righetti C., Spirito A., Luciano A., Destefanis P., Nangeroni G., Angusti T., Anselmino M. (2021). The prevalence of TTR cardiac amyloidosis among patients undergoing bone scintigraphy. J. Nucl. Cardiol..

[19] Suomalainen O., Pilv J., Loimaala A., Mätzke S., Heliö T., Uusitalo V. (2022). Prognostic significance of incidental suspected transthyretin amyloidosis on routine bone scintigraphy. J. Nucl. Cardiol..

[20] Cuscaden C., Ramsay S.C., Prasad S., Goodwin B., Smith J. (2021). Estimation of prevalence of transthyretin (ATTR) cardiac amyloidosis in an Australian subpopulation using bone scans with echocardiography and clinical correlation. J. Nucl. Cardiol..

[21] Kim H.M., Sohn D.W., Paeng J.C. (2019). Prevalence of Positive ^99m^Tc-DPD Scintigraphy as an Indicator of the Prevalence of Wild-type Transthyretin Amyloidosis in the Elderly. Int. Heart J..

[22] Mohamed-Salem L., Santos-Mateo J.J., Sanchez-Serna J., Hernández-Vicente Á., Reyes-Marle R., Castellón Sánchez M.I., Claver-Valderas M.A., Gonzalez-Vioque E., Haro-Del Moral F.J., García-Pavía P. (2018). Prevalence of wild type ATTR assessed as myocardial uptake in bone scan in the elderly population. Int. J. Cardiol..

[23] Longhi S., Guidalotti P.L., Quarta C.C., Gagliardi C., Milandri A., Lorenzini M., Potena L., Leone O., Bartolomei I., Pastorelli F. (2014). Identification of TTR-related subclinical amyloidosis with ^99m^Tc-DPD scintigraphy. JACC Cardiovasc. Imaging.

[24] Al-Nahhas A.M., Jinnouchi S., Anagnostopoulos C., Hirsch W., Heary T., McCready V.R. (1995). Clinical significance of technetium-99m methylene diphosphonate myocardial uptake: Association with carcinoma of the prostate. Eur. J. Nucl. Med..

[25] Damy T., Kristen A.V., Suhr O.B., Maurer M.S., Planté-Bordeneuve V., Yu C.R., Ong M.L., Coelho T., Rapezzi C., THAOS Investigators (2019). Transthyretin cardiac amyloidosis in continental Western Europe: An insight through the Transthyretin Amyloidosis Outcomes Survey (THAOS). Eur. Heart J..

[26] Obi C.A., Mostertz W.C., Griffin J.M., Judge D.P. (2022). ATTR Epidemiology, Genetics, and Prognostic Factors. Methodist Debakey Cardiovasc. J..

[27] Mejia Baranda J., Ljungberg J., Wixner J., Anan I., Oskarsson V. (2022). Epidemiology of hereditary transthyretin amyloidosis in the northernmost region of Sweden: A retrospective cohort study. Amyloid.

[28] Maurer M.S., Hanna M., Grogan M., Dispenzieri A., Witteles R., Drachman B., Judge D.P., Lenihan D.J., Gottlieb S.S., Shah S.J. (2016). THAOS Investigators. Genotype and Phenotype of Transthyretin Cardiac Amyloidosis: THAOS (Transthyretin Amyloid Outcome Survey). J. Am. Coll. Cardiol..

[29] Delbarre M.A., Girardon F., Roquette L., Blanc-Durand P., Hubaut M.A., Hachulla É., Semah F., Huglo D., Garcelon N., Marchal E. (2023). Deep Learning on Bone Scintigraphy to Detect Abnormal Cardiac Uptake at Risk of Cardiac Amyloidosis. JACC Cardiovasc. Imaging.

[30] Castiglione V., Aimo A., Todiere G., Barison A., Fabiani I., Panichella G., Genovesi D., Bonino L., Clemente A., Cademartiri F. (2023). Role of Imaging in Cardiomyopathies. Card. Fail. Rev..

[31] Gherghe M., Lazar A.M., Sterea M.C., Spiridon P.M., Motas N., Gales L.N., Coriu D., Badelita S.N., Mutuleanu M.D. (2023). Quantitative SPECT/CT Parameters in the Assessment of Transthyretin Cardiac Amyloidosis-A New Dimension of Molecular Imaging. J. Cardiovasc. Dev. Dis..

[32] Slart R.H.J.A., Chen W., Tubben A., Tingen H.S.A., Davies D.R., Grogan M., Wechalekar A.D., Kittleson M.M., Thomson L.E.J., Slomka P.J. (2023). Emerging Role of Scintigraphy Using Bone-Seeking Tracers for Diagnosis of Cardiac Amyloidosis: AJR Expert Panel Narrative Review. AJR Am. J. Roentgenol..

[33] Shetty M., Malhotra S. (2023). Novel Tracers for the Imaging of Cardiac Amyloidosis. J. Nucl. Med. Technol..

[34] Waheed A., Dorbala S. (2023). Current Status of Radionuclide Imaging of Transthyretin Cardiac Amyloidosis. Cardiol. Clin..

[35] Caponetti A.G., Accietto A., Saturi G., Ponziani A., Sguazzotti M., Massa P., Giovannetti A., Ditaranto R., Parisi V., Leone O. (2023). Screening approaches to cardiac amyloidosis in different clinical settings: Current practice and future perspectives. Front. Cardiovasc. Med..

[36] Wieczorek E., Ożyhar A. (2021). Transthyretin: From Structural Stability to Osteoarticular and Cardiovascular Diseases. Cells.

