# Supplementary material for: Prevalence of Incidental Findings Suspicious for Transthyretin Cardiac Amyloidosis among Patients Undergoing Bone Scintigraphy: A Systematic Review and a Meta-Analysis
Source: J Clin Med. 2023 Sep 1;12(17):5698. doi: 10.3390/jcm12175698 (PMC10488435; doi:10.3390/jcm12175698)
Supplement: Supplementary file 1 [file jcm-12-05698-s001.zip › Table S2. List of screened records.pdf]

List of records with final decision on inclusion or exclusion in the systematic review and meta-analysis

1: Youssef ME, Yahya G, Popoviciu MS, Cavalu S, Abd-Eldayem MA, Saber S. Unlocking the Full Potential of SGLT2 Inhibitors: Expanding Applications beyond Glycemic Control. Int J Mol Sci. 2023 Mar 23;24(7):6039. doi: 10.3390/ijms24076039.
Excluded (reason: outside the field of interest)

2: Navarro-Saez MDC, Feijoo-Massó C, Bravo Ferrer ZDC, Oliva Morera JC, Balado González AM, Palau-Domínguez A, Guillamon Toran L, Comet Monte R, Fernández-Codina A. Trends in diagnosis of cardiac transthyretin amyloidosis: 3-year analysis of scintigraphic studies: Prevalence of myocardial uptake and its predictor factors. Int J Cardiovasc Imaging. 2023 Apr 1. doi: 10.1007/s10554-023-02840-y.
Included in the systematic review and meta-analysis

3: Cersosimo A, Bonelli A, Lombardi CM, Moreo A, Pagnesi M, Tomasoni D, Arabia G, Vizzardi E, Adamo M, Farina D, Metra M, Inciardi RM. Multimodality imaging in the diagnostic management of concomitant aortic stenosis and transthyretin-related wild-type cardiac amyloidosis. Front Cardiovasc Med. 2023 Mar 14;10:1108696. doi: 10.3389/fcvm.2023.1108696.
Excluded (reason: outside the field of interest)

4: Ruiz-Hueso R, Salamanca-Bautista P, Quesada-Simón MA, Yun S, Conde-Martel A, Morales-Rull JL, Suárez-Gil R, García-García JÁ, Llàcer P, Fonseca-Aizpuru EM, Amores-Arriaga B, Martínez-González Á, Armengou-Arxe A, Peña-Somovilla JL, López-Reboiro ML, Aramburu-Bodas Ó; PREVAMIC Investigators Group. Estimating the Prevalence of Cardiac Amyloidosis in Old Patients with Heart Failure-Barriers and Opportunities for Improvement: The PREVAMIC Study. J Clin Med. 2023 Mar 15;12(6):2273. doi: 10.3390/jcm12062273.
Excluded (reason: outside the field of interest)

5: Rauf MU, Hawkins PN, Cappelli F, Perfetto F, Zampieri M, Argiro A, Petrie A, Law S, Porcari A, Razvi Y, Bomsztyk J, Ravichandran S, Ioannou A, Patel R, Starr N, Hutt DF, Mahmood S, Wisniowski B, Martinez-Naharro A, Venneri L, Whelan C, Roczenio D, Gilbertson J, Lachmann HJ, Wechalekar AD, Rapezzi C, Serenelli M, Massa P, Caponetti AG, Ponziani A, Accietto A, Giovannetti A, Saturi G, Sguazzotti M, Gagliardi C, Biagini E, Longhi S, Fontana M, Gillmore JD. Tc-99m labelled bone scintigraphy in suspected cardiac amyloidosis. Eur Heart J. 2023 Mar 22:ehad139. doi: 10.1093/eurheartj/ehad139.
Excluded (reason: outside the field of interest)

6: Tsushima T, Terao T, Narita K, Fukumoto A, Ikeda D, Kamura Y, Kuzume A, Tabata R, Miura D, Takeuchi M, Matsue K. Clinical Characteristics and Outcomes of Cyclin D1-Positive AL Amyloidosis. Am J Clin Pathol. 2023 Mar 20:aqad013. doi: 10.1093/ajcp/aqad013.
Excluded (reason: outside the field of interest)

7: Raval M, Siddiq S, Sharma K, Sanghvi L, Jain A, Patel S, Trivedi J, Uttam Chandani K, Patel D, Desai R. A review of recent advances in the diagnosis of cardiac amyloidosis, treatment of its cardiac complications, and disease-modifying therapies. F1000Res. 2023 Feb 20;12:192. doi: 10.12688/f1000research.130285.1.
Excluded (reason: outside the field of interest)

8: Nuzzi V, Porcari A, Gigli M, Zaja F, Dore F, Bussani R, Sinagra G, Merlo M. A case report of isolated cardiac light chain amyloidosis without clinically overt heart failure: an under-recognized presentation. Eur Heart J Case Rep. 2023 Feb 13;7(3):ytad072. doi: 10.1093/ehjcr/ytad072. PMID: 36909839; PMCID: PMC9998033.
Excluded (reason: outside the field of interest)

9: Naiki H, Yamaguchi A, Sekijima Y, Ueda M, Ohashi K, Hatakeyama K, Ikeda Y, Hoshii Y, Shintani-Domoto Y, Miyagawa-Hayashino A, Tsujikawa H, Endo J, Arai T, Ando Y. Steep increase in the number of transthyretin-positive cardiac biopsy cases in Japan: evidence obtained by the nation-wide pathology consultation for the typing diagnosis of amyloidosis. Amyloid. 2023 Feb 16:1-6. doi: 10.1080/13506129.2023.2180334.

Excluded (reason: outside the field of interest)

10: Le Bras P, Plard L, Le Gouill C, Piriou N, Touchefeu Y. Transthyretin Cardiac Amyloidosis Mimicking Immune Checkpoint-Induced Myocarditis in a Patient Treated with Atezolizumab and Bevacizumab for Advanced Hepatocellular Carcinoma: A Case Report. Case Rep Oncol. 2022 Nov 8;15(3):967-973. doi: 10.1159/000526534.
Excluded (reason: outside the field of interest)

11: Testa S, Kumar J, Goodell AJ, Zehnder JL, Alexander KM, Sidana S, Arai S, Witteles RM, Liedtke M. Prevalence, mutational spectrum and clinical implications of clonal hematopoiesis of indeterminate potential in plasma cell dyscrasias. Semin Oncol. 2022 Dec;49(6):465-475. doi: 10.1053/j.seminoncol.2022.11.001.
Excluded (reason: outside the field of interest)

12: Schneider SM, Kochar K, Ruge M, Marek-Iannucci S, Datta T, Hajduczok A, Ullah W, Rajapreyar I, Brailovsky Y. Cardiogenic Shock Due to Atrial Arrhythmia as the Initial Presentation of Transthyretin Cardiac Amyloidosis. JACC Case Rep. 2022 Nov 16;4(22):1490-1495. doi: 10.1016/j.jaccas.2022.07.041.
Excluded (reason: outside the field of interest)

13: Yadav P, Lee YH, Panday H, Kant S, Bajwa N, Parashar R, Jha SK, Jha NK, Nand P, Lee SS, Jha AK. Implications of Microorganisms in Alzheimer's Disease. Curr Issues Mol Biol. 2022 Sep 30;44(10):4584-4615. doi: 10.3390/cimb44100314.
Excluded (reason: outside the field of interest)

14: Porcari A, Hutt DF, Grigore SF, Quigley AM, Rowczenio D, Gilbertson J, Patel R, Razvi Y, Ioannou A, Rauf MU, Martinez-Naharro A, Venneri L, Hawkins PN, Fontana M, Gillmore JD. Comparison of different technetium-99m-labelled bone tracers for imaging cardiac amyloidosis. Eur J Prev Cardiol. 2023 Feb 14;30(3):e4-e6. doi: 10.1093/eurjpc/zwac237.
Excluded (reason: outside the field of interest)

15: Jain A, Zahra F. Transthyretin Amyloid Cardiomyopathy (ATTR-CM). 2022 Sep 26. In: StatPearls [Internet]. Treasure Island (FL): StatPearls Publishing; 2023 Jan–.
Excluded (reason: outside the field of interest)

16: Yang H, Li R, Ma F, Wei Y, Liu Y, Sun Y, He X, Zeng H, Yan J, Wang DW, Wang H. An echo score raises the suspicion of cardiac amyloidosis in Chinese with heart failure with preserved ejection fraction. ESC Heart Fail. 2022 Dec;9(6):4280-4290. doi: 10.1002/ehf2.14164.
Excluded (reason: outside the field of interest)

17: Wang J, Yang S, Liao P, Zeng L, Ling W, Wan L, Weng J, Zhong L. Incidence and effect of secondary cardiac amyloidosis on outcomes of patients with t(11;14) multiple myeloma. Front Cardiovasc Med. 2022 Sep 2;9:994384. doi: 10.3389/fcvm.2022.994384.
Excluded (reason: outside the field of interest)

18: Ge Y, Pandya A, Cuddy SAM, Singh A, Singh A, Dorbala S. Modeling the Cost and Health Impacts of Diagnostic Strategies in Patients with Suspected Transthyretin Cardiac Amyloidosis. J Am Heart Assoc. 2022 Sep 20;11(18):e026308. doi: 10.1161/JAHA.122.026308. Epub 2022 Sep 14.
Excluded (reason: outside the field of interest)

19: Tzagournissakis M, Foukarakis E, Samonakis D, Tsilimbaris M, Michaelidou K, Mathioudakis L, Marinis A, Giannakoudakis E, Spanaki C, Skoula I, Erimaki S, Amoiridis G, Koutsis G, Koukouraki S, Stylianou K, Plaitakis A, Mitsias PD, Zaganas I. High Hereditary Transthyretin-Related Amyloidosis Prevalence in Crete: Genetic Heterogeneity and Distinct Phenotypes. Neurol Genet. 2022 Sep 9;8(5):e200013. doi: 10.1212/NXG.0000000000200013.
Excluded (reason: outside the field of interest)

20: Davies DR, Redfield MM, Scott CG, Minamisawa M, Grogan M, Dispenzieri A, Chareonthaitawee P, Shah AM, Shah SJ, Wehbe RM, Solomon SD, Reddy YNV, Borlaug BA, AbouEzzeddine OF. A Simple Score to Identify Increased Risk of Transthyretin Amyloid Cardiomyopathy in Heart Failure With Preserved Ejection Fraction. JAMA Cardiol. 2022 Oct 1;7(10):1036-1044. doi: 10.1001/jamacardio.2022.1781.
Excluded (reason: outside the field of interest)

21: Westin O, Fosbøl EL, Maurer MS, Leicht BP, Hasbak P, Mylin AK, Rørvig S, Lindkær TH, Johannesen HH, Gustafsson F. Screening for Cardiac Amyloidosis 5 to 15 Years After Surgery for Bilateral Carpal Tunnel Syndrome. J Am Coll Cardiol. 2022 Sep 6;80(10):967-977. doi: 10.1016/j.jacc.2022.06.026.
Excluded (reason: outside the field of interest)

22: Donà C, Nitsche C, Koschutnik M, Heitzinger G, Mascherbauer K, Kammerlander AA, Dannenberg V, Halavina K, Rettl R, Duca F, Traub-Weidinger T, Puchinger J, Gunacker PC, Lamm G, Vock P, Lileg B, Philipp V, Staudenherz A, Calabretta R, Hacker M, Agis H, Bartko P, Hengstenberg C, Fontana M, Goliasch G, Mascherbauer J. Unveiling Cardiac Amyloidosis, its Characteristics, and Outcomes Among Patients With MR Undergoing Transcatheter Edge-to-Edge MV Repair. JACC Cardiovasc Interv. 2022 Sep 12;15(17):1748-1758. doi: 10.1016/j.jcin.2022.06.009.
Excluded (reason: outside the field of interest)

23: Porcari A, Fontana M, Gillmore JD. Transthyretin cardiac amyloidosis. Cardiovasc Res. 2023 Feb 3;118(18):3517-3535. doi: 10.1093/cvr/cvac119.
Excluded (reason: outside the field of interest)

24: Ramsell S, Arias Bermudez C, Takem Baiyee CAM, Rodgers B, Parikh S, Almaani S, Sharma N, LoRusso S, Freimer M, Redder E, Bumma N, Vallkati A, Efebera Y, Kahwash R, Campbell CM. Beta-Adrenergic Antagonist Tolerance in Amyloid Cardiomyopathy. Front Cardiovasc Med. 2022 Jul 11;9:907597. doi: 10.3389/fcvm.2022.907597.
Excluded (reason: outside the field of interest)

25: Rossi M, Varrà GG, Porcari A, Saro R, Pagura L, Lalario A, Dore F, Bussani R, Sinagra G, Merlo M. Re-Definition of the Epidemiology of Cardiac Amyloidosis. Biomedicines. 2022 Jun 30;10(7):1566. doi: 10.3390/biomedicines10071566. PMID: 35884871; PMCID: PMC9313045.
Excluded (reason: review)

26: Gao YJ, Shen KN, Chang L, Feng J, Mao YY, Zhang L, Cao XX, Zhou DB, Li J. Prevalence, clinical characteristics and treatment outcome of factor X deficiency in a consecutive cohort of primary light-chain amyloidosis. Leuk Res. 2022 Sep;120:106917. doi: 10.1016/j.leukres.2022.106917.
Excluded (reason: outside the field of interest)

27: Nitsche C, Mascherbauer K, Calabretta R, Koschutnik M, Dona C, Dannenberg V, Hofer F, Halavina K, Kammerlander AA, Traub-Weidinger T, Goliasch G, Hengstenberg C, Hacker M, Mascherbauer J. Prevalence and Outcomes of Cardiac Amyloidosis in All-Comer Referrals for Bone Scintigraphy. J Nucl Med. 2022 Dec;63(12):1906-1911. doi: 10.2967/jnumed.122.264041.
Included in the systematic review and meta-analysis

28: Lizarazo Ortega DA, Valderrama BP, González-Robledo G, Trujillo PB. A Case of Wild-type Cardiac Transthyretin Amyloidosis Diagnosed by Non-invasive Methods. Curr Med Imaging. 2023;19(4):402-406. doi: 10.2174/1573405618666220610091446.
Excluded (reason: outside the field of interest)

29: Porcari A, Rossi M, Dore F, Imazio M, Fontana M, Merlo M, Sinagra G. I dieci punti che il cardiologo deve conoscere su scintigrafia miocardica con traccianti ossei, amiloidosi e cuore [Ten questions for the cardiologist about

cardiac scintigraphy with bone tracers, amyloidosis and the heart]. G Ital Cardiol (Rome). 2022 Jun;23(6):424-432. Italian. doi: 10.1714/3810.37938.
Excluded (reason: outside the field of interest)

30: Wang M, Pan W, Xu Y, Zhang J, Wan J, Jiang H. Microglia-Mediated Neuroinflammation: A Potential Target for the Treatment of Cardiovascular Diseases. J Inflamm Res. 2022 May 25;15:3083-3094. doi: 10.2147/JIR.S350109.
Excluded (reason: outside the field of interest)

31: Hussain M, Krywanczyk A, Donnellan E, Martyn T, Hassan OA, Alkharabsheh S, Watson C, Tang WH, Kwon D, Cremer P, Cheng F, Kanj M, Griffin B, Tan C, Rodriguez ER, Hanna M, Jaber W, Collier P. Association Between Atrial Uptake on Cardiac Scintigraphy With Technetium-99m-Pyrophosphate Labeled Bone-Seeking Tracers and Atrial Fibrillation. Circ Cardiovasc Imaging. 2022 May;15(5):e013829. doi: 10.1161/CIRCIMAGING.121.013829.
Excluded (reason: outside the field of interest)

32: Halme HL, Ihalainen T, Suomalainen O, Loimaala A, Mätzke S, Uusitalo V, Sipilä O, Hippeläinen E. Convolutional neural networks for detection of transthyretin amyloidosis in 2D scintigraphy images. EJNMMI Res. 2022 May 7;12(1):27. doi: 10.1186/s13550-022-00897-9.
Included in the systematic review but not in the meta-analysis (for possible patient fata overlap with another study)

33: Aimo A, Merlo M, Porcari A, Georgiopoulos G, Pagura L, Vergaro G, Sinagra G, Emdin M, Rapezzi C. Redefining the epidemiology of cardiac amyloidosis. A systematic review and meta-analysis of screening studies. Eur J Heart Fail. 2022 Dec;24(12):2342-2351. doi: 10.1002/ejhf.2532.
Excluded (reason: review)

34: Salvalaggio A, Cipriani A, Righetto S, Artioli P, Sinigiani G, De Michieli L, Cason M, Pilichou K, Cecchin D, Briani C. Incidental cardiac uptake of ^{99m}Tc-diphosphonates is predictive of poor outcome: data from 9616 bone scintigraphies. J Nucl Cardiol. 2022 Dec;29(6):3419-3425. doi: 10.1007/s12350-022-02961-2.
Included in the systematic review and in the meta-analysis

35: Gill SS, Fellin E, Stampke L, Zhao Y, Masri A. Clinical Clues and Diagnostic Workup of Cardiac Amyloidosis. Methodist Debakey Cardiovasc J. 2022 Mar 14;18(2):36-46. doi: 10.14797/mdcvj.1061.
Excluded (reason: outside the field of interest)

36: Xu J, Qiu Z, Yan M, Wang B, Song Z, Liu H, Wang M, Cen X. Prognostic Factors of AL-PCMM and AL-MM: A Single-Center Retrospective Study. Int J Med Sci. 2022 Mar 14;19(3):588-595. doi: 10.7150/ijms.61712.
Excluded (reason: outside the field of interest)

37: Rapezzi C, Aimo A, Serenelli M, Barison A, Vergaro G, Passino C, Panichella G, Sinagra G, Merlo M, Fontana M, Gillmore J, Quarta CC, Maurer MS, Kittleson MM, Garcia-Pavia P, Emdin M. Critical Comparison of Documents From Scientific Societies on Cardiac Amyloidosis: JACC State-of-the-Art Review. J Am Coll Cardiol. 2022 Apr 5;79(13):1288-1303.
Excluded (reason: outside the field of interest)

38: Barge-Caballero G, Barge-Caballero E, López-Pérez M, Bilbao-Quesada R, González-Babarro E, Gómez-Otero I, López-López A, Gutiérrez-Feijoo M, Varela-Román A, González-Juanatey C, Díaz-Castro Ó, Crespo-Leiro MG. Syncope in patients with transthyretin amyloid cardiomyopathy: clinical features and outcomes. Postgrad Med. 2022 May;134(4):420-428. doi: 10.1080/00325481.2022.2054174.
Excluded (reason: outside the field of interest)

39: Stan C, Mititelu R, Adam RD, Jurcuţ R. Awareness of Nuclear Medicine Physicians in Romania Regarding the Diagnostic of Cardiac Amyloidosis-A Survey- Based Study. Diagnostics (Basel). 2022 Feb 21;12(2):556. doi: 10.3390/diagnostics12020556.
Excluded (reason: outside the field of interest)

40: Chiu A, Dasari S, Kurtin PJ, Theis JD, Vrana JA, Dispenzieri A, Rech KL, Dao LN, Howard MT, Grogan M, McPhail ED. Bone marrow amyloid: a comprehensive analysis of 1,469 samples, including amyloid type, clinical features, and morphologic distribution. Amyloid. 2022 Sep;29(3):156-164. doi: 10.1080/13506129.2022.2031963.
Excluded (reason: outside the field of interest)

41: Takashio S, Tokitsu T, Matsukawa M, Sakaino N, Fujimoto K, Sakamoto T, Noda K, Tsunoda R, Misumi I, Hirai N, Doi H, Koide S, Mizuno Y, Hirose T, Kurokawa H, Kajiwara I, Ohba K, Miyamoto S, Araki S, Yamamoto E, Matsushita K, Ueda M, Tsujita K; Kumamoto Cardiac Amyloid Survey Investigators. Incidence, clinical characteristics, and diagnostic approach in transthyretin amyloid cardiomyopathy: The Kumamoto Cardiac Amyloidosis Survey. J Cardiol. 2022 Jul;80(1):49-55. doi: 10.1016/j.jjcc.2022.01.002.
Excluded (reason: outside the field of interest)

42: Uusitalo V, Suomalainen O, Loimaala A, Mätzke S, Heliö T. Prognostic Value of ^{99m}Tc-HMDP Scintigraphy in Elderly Patients With Chronic Heart Failure. Heart Lung Circ. 2022 May;31(5):629-637. doi: 10.1016/j.hlc.2021.11.018.
Excluded (reason: outside the field of interest)

43: Bourque JM, Schepart A, Bhambri R, Castaño A, O'Brien A, Chen Y, Prasad S, Roy A, Grodin JL. Temporal Trends in Diagnostic Testing Patterns for Wild-Type Transthyretin Amyloid Cardiomyopathy in the Medicare Fee-for-Service Population. Am J Cardiol. 2022 Mar 15;167:98-103. doi: 10.1016/j.amjcard.2021.11.048.
Excluded (reason: outside the field of interest)

44: Abdelazeem B, Manasrah N, Yousaf A, Gjeka R, Kunadi A. Light Chain (AL) Cardiac Amyloidosis: A Diagnostic Dilemma. Cureus. 2021 Nov 5;13(11):e19278. doi: 10.7759/cureus.19278.
Excluded (reason: outside the field of interest)

45: Singal AK, Bansal R, Singh A, Dorbala S, Sharma G, Gupta K, Saxena A, Bhargava B, Karthikeyan G, Ramakrishnan S, Bisoi AK, Hote MP, Rajashekar P, Chowdhury UK, Devagourou V, Patel C, Ray R, Arawa SK, Mishra S. Concomitant Transthyretin Amyloidosis and Severe Aortic Stenosis in Elderly Indian Population: A Pilot Study. JACC CardioOncol. 2021 Oct 19;3(4):565-576. doi: 10.1016/j.jaccao.2021.08.008.
Excluded (reason: outside the field of interest)

46: Korosoglou G, Giusca S, André F, Aus dem Siepen F, Nunninger P, Kristen AV, Frey N. Diagnostic Work-Up of Cardiac Amyloidosis Using Cardiovascular Imaging: Current Standards and Practical Algorithms. Vasc Health Risk Manag. 2021 Oct 23;17:661-673. doi: 10.2147/VHRM.S295376.
Excluded (reason: outside the field of interest)

47: Bäcker HC, Galle SE, Lentzsch S, Freibott CE, Shoap S, Strauch RJ, Rosenwasser MP. Flexor tenosynovectomy in carpal tunnel syndrome as a screening tool for early diagnosis of amyloidosis. Ir J Med Sci. 2022 Oct;191(5):2427-2430. doi: 10.1007/s11845-021-02832-8.
Excluded (reason: outside the field of interest)

48: Shimoni S, Zikri M, Haberman D, Livschitz S, Tshori S, Fabricant Y, Meledin V, Gandelman G, Goland S, George J. Transthyretin cardiac amyloidosis in patients after TAVR: clinical and echocardiographic findings and long term survival. ESC Heart Fail. 2021 Dec;8(6):4549-4561. doi: 10.1002/ehf2.13667.
Excluded (reason: outside the field of interest)

49: Fabbri G, Serenelli M, Cantone A, Sanguettoli F, Rapezzi C. Transthyretin amyloidosis in aortic stenosis: clinical and therapeutic implications. Eur Heart J Suppl. 2021 Oct 8;23(Suppl E):E128-E132. doi: 10.1093/eurheartj/suab107.
Excluded (reason: outside the field of interest)

50: Nguyen FD, Rodriguez M, Krittanawong C, Witteles R, Lenihan DJ. Misconceptions and Facts About Cardiac Amyloidosis. Am J Cardiol. 2021 Dec 1;160:99-105. doi: 10.1016/j.amjcard.2021.08.045.
Excluded (reason: outside the field of interest)

51: Roshankar G, White GC, Cadet S, Fine NM, Chan D, White JA, Jimenez-Zepeda V, Slomka PJ, Miller RJH. Quantitative technetium pyrophosphate and cardiovascular magnetic resonance in patients with suspected cardiac amyloidosis. J Nucl Cardiol. 2022 Oct;29(5):2679-2690. doi: 10.1007/s12350-021-02806-4.
Excluded (reason: outside the field of interest)

52: Bonelli A, Paris S, Nardi M, Henein MY, Agricola E, Troise G, Faggiano P. Aortic Valve Stenosis and Cardiac Amyloidosis: A Misleading Association. J Clin Med. 2021 Sep 18;10(18):4234. doi: 10.3390/jcm10184234.
Excluded (reason: outside the field of interest)

53: Patel KP, Scully PR, Nitsche C, Kammerlander AA, Joy G, Thornton G, Hughes R, Williams S, Tillin T, Captur G, Chacko L, Kelion A, Sabharwal N, Newton JD, Kennon S, Ozkor M, Mullen M, Hawkins PN, Gillmore JD, Menezes L, Pugliese F, Hughes AD, Fontana M, Lloyd G, Treibel TA, Mascherbauer J, Moon JC. Impact of afterload and infiltration on coexisting aortic stenosis and transthyretin amyloidosis. Heart. 2022 Jan;108(1):67-72. doi: 10.1136/heartjnl-2021-319922.
Excluded (reason: outside the field of interest)

54: AbouEzzeddine OF, Davies DR, Scott CG, Fayyaz AU, Askew JW, McKie PM, Noseworthy PA, Johnson GB, Dunlay SM, Borlaug BA, Chareonthaitawee P, Roger VL, Dispenzieri A, Grogan M, Redfield MM. Prevalence of Transthyretin Amyloid Cardiomyopathy in Heart Failure With Preserved Ejection Fraction. JAMA Cardiol. 2021 Nov 1;6(11):1267-1274. doi: 10.1001/jamacardio.2021.3070.
Excluded (reason: outside the field of interest)

55: Dayco J, Weaver M, Sumbal N, Theisen R, Raheem S. Ruptured Bullae: A Case of Transthyretin Cardiac Amyloidosis. Cureus. 2021 Jul 11;13(7):e16318. doi: 10.7759/cureus.16318.
Excluded (reason: outside the field of interest)

56: Tini G, Sessarego E, Benenati S, Vianello PF, Musumeci B, Autore C, Canepa M. Yield of bone scintigraphy screening for transthyretin-related cardiac amyloidosis in different conditions: Methodological issues and clinical implications. Eur J Clin Invest. 2021 Dec;51(12):e13665. doi: 10.1111/eci.13665.
Excluded (reason: outside the field of interest)

57: Oye M, Dhruva P, Kandah F, Oye M, Missov E. Cardiac amyloid presenting as cardiogenic shock: case series. Eur Heart J Case Rep. 2021 Jul 26;5(7):ytab252. doi: 10.1093/ehjcr/ytab252.
Excluded (reason: outside the field of interest)

58: Melero Polo J, Roteta Unceta-Barrenechea A, Revilla Martí P, Pérez-Palacios R, Gracia Gutiérrez A, Bueno Juana E, Andrés Gracia A, Atienza Ayala S, Aibar Arregui MÁ. Echocardiographic markers of cardiac amyloidosis in patients with heart failure and left ventricular hypertrophy. Cardiol J. 2023;30(2):266-275. doi: 10.5603/CJ.a2021.0085.
Excluded (reason: outside the field of interest)

59: Pan Y, Wang Y, Zhou Y, Xi Y, Zhang Y. 99mTc-Pyrophosphate Retention in Atelectatic Pulmonary Tissues of a Patient With Transthyretin Cardiac Amyloidosis. Clin Nucl Med. 2021 Oct 1;46(10):820-821. doi: 10.1097/RLU.0000000000003816.
Excluded (reason: outside the field of interest)

60: Godara A, Riesenburger RI, Zhang DX, Varga C, Fogaren T, Siddiqui NS, Yu A, Wang A, Mastroianni M, Dowd R, Nail TJ, McPhail ED, Kurtin PJ, Theis JD, Toskic D, Arkun K, Pilichowska M, Kryzanski J, Patel AR, Comenzo R.

Association between spinal stenosis and wild-type ATTR amyloidosis. Amyloid. 2021 Dec;28(4):226-233. doi: 10.1080/13506129.2021.1950681.
Excluded (reason: outside the field of interest)

61: Goland S, Volodarsky I, Fabricant Y, Livschitz S, Tshori S, Cuciuc V, Zilberman L, Fugenfirov I, Meledin V, Shimoni S, Josfberg S, George J. Wild-type TTR amyloidosis among patients with unexplained heart failure and systolic LV dysfunction. PLoS One. 2021 Jul 9;16(7):e0254104. doi: 10.1371/journal.pone.0254104.
Excluded (reason: outside the field of interest)

62: Inomata T, Tahara N, Nakamura K, Endo J, Ueda M, Ishii T, Kitano Y, Koyama J. Diagnosis of wild-type transthyretin amyloid cardiomyopathy in Japan: red-flag symptom clusters and diagnostic algorithm. ESC Heart Fail. 2021 Aug;8(4):2647-2659. doi: 10.1002/ehf2.13473.
Excluded (reason: outside the field of interest)

63: Sugiura K, Kozuki H, Ueba H, Kubo T, Ochi Y, Baba Y, Miyagawa K, Noguchi T, Hirota T, Yamasaki N, Wada N, Nakashima J, Murakami I, Ikeuchi M, Kitaoka H. Tenosynovial and Cardiac Transthyretin Amyloidosis in Japanese Patients Undergoing Carpal Tunnel Release. Circ Rep. 2021 May 27;3(6):338-344. doi: 10.1253/circrep.CR-21-0046.
Excluded (reason: outside the field of interest)

64: Delaney FT, Dempsey P, Welaratne I, Buckley B, O'Sullivan D, O'Connell M. Incidental cardiac uptake in bone scintigraphy: increased importance and association with cardiac amyloidosis. BJR Case Rep. 2021 Jan 19;7(3):20200161. doi: 10.1259/bjrcr.20200161.
Excluded (reason: small case series)

65: Sood RF, Kamenko S, McCreary E, Sather BK, Schmitt M, Peterson SL, Lipira AB. Diagnosing Systemic Amyloidosis Presenting as Carpal Tunnel Syndrome: A Risk Nomogram to Guide Biopsy at Time of Carpal Tunnel Release. J Bone Joint Surg Am. 2021 Jul 21;103(14):1284-1294. doi: 10.2106/JBJS.20.02093.
Excluded (reason: outside the field of interest)

66: Paeng JC, Choi JY. Nuclear Imaging for Cardiac Amyloidosis: Bone Scan, SPECT/CT, and Amyloid-Targeting PET. Nucl Med Mol Imaging. 2021 Apr;55(2):61-70. doi: 10.1007/s13139-020-00681-4.
Excluded (reason: outside the field of interest)

67: Devesa A, Camblor Blasco A, Pello Lázaro AM, Askari E, Lapeña G, Gómez Talavera S, Taibo Urquía M, Rodríguez Olleros C, Tuñón J, Ibáñez B, Aceña Á. Prevalence of transthyretin amyloidosis in patients with heart failure and no left ventricular hypertrophy. ESC Heart Fail. 2021 Aug;8(4):2856-2865. doi: 10.1002/ehf2.13360.
Excluded (reason: outside the field of interest)

68: Addison D, Slivnick JA, Campbell CM, Vallakati A, Jneid H, Schelbert E. Recent Advances and Current Dilemmas in the Diagnosis and Management of Transthyretin Cardiac Amyloidosis. J Am Heart Assoc. 2021 May 4;10(9):e019840. doi: 10.1161/JAHA.120.019840.
Excluded (reason: outside the field of interest)

69: Tsai K, Yu AC, Livhits MJ, Sajed D, Leung AM, Cheung DS. Systemic light-chain amyloidosis incidentally diagnosed after subtotal parathyroidectomy and thyroid lobectomy. BMJ Case Rep. 2021 Apr 19;14(4):e241282. doi: 10.1136/bcr-2020-241282.
Excluded (reason: outside the field of interest)

70: Nijst P, Tang WW. Recent advances in the diagnosis and management of amyloid cardiomyopathy. Fac Rev. 2021 Mar 24;10:31. doi: 10.12703/r/10-31.
Excluded (reason: outside the field of interest)

71: Bianco M, Parente A, Biolè C, Righetti C, Spirito A, Luciano A, Destefanis P, Nangeroni G, Angusti T, Anselmino M, Montagna L. The prevalence of TTR cardiac amyloidosis among patients undergoing bone scintigraphy. J Nucl Cardiol. 2021 Jun;28(3):825-830. doi: 10.1007/s12350-021-02575-0.
Included in the systematic review and meta-analysis

72: Abdallah N, Muchtar E, Dispenzieri A, Gonsalves W, Buadi F, Lacy MQ, Hayman SR, Kourelis T, Kapoor P, Go RS, Warsame R, Leung N, Rajkumar SV, Kyle RA, Pruthi RK, Gertz MA, Kumar SK. Coagulation Abnormalities in Light Chain Amyloidosis. Mayo Clin Proc. 2021 Feb;96(2):377-387. doi: 10.1016/j.mayocp.2020.06.061.
Excluded (reason: outside the field of interest)

73: Bistola V, Parissis J, Foukarakis E, Valsamaki PN, Anastasakis A, Koutsis G, Efthimiadis G, Kastritis E. Practical recommendations for the diagnosis and management of transthyretin cardiac amyloidosis. Heart Fail Rev. 2021 Jul;26(4):861-879. doi: 10.1007/s10741-020-10062-w.
Excluded (reason: outside the field of interest)

74: Chandrashekar P, Masri A. Incidental extra-cardiac uptake of ^{99m}Tc-pyrophosphate. J Nucl Cardiol. 2022 Oct;29(5):2721-2723. doi: 10.1007/s12350-020-02498-2.
Excluded (reason: outside the field of interest)

75: Tanaka H, Hosono M, Kanagaki M, Shimizu M, Matsubara N, Kawabata K, Miyamoto T, Itoi K. A case of cardiac amyloidosis incidentally detected by bone scintigraphy. Asia Ocean J Nucl Med Biol. 2021 Winter;9(1):71-75. doi: 10.22038/AOJNMB.2020.50508.1350.
Excluded (reason: case report)

76: Vianello PF, La Malfa G, Tini G, Mazzola V, Miceli A, Santolini E, Briano S, Porto I, Canepa M. Prevalence of transthyretin amyloid cardiomyopathy in male patients who underwent bilateral carpal tunnel surgery: The ACTUAL study. Int J Cardiol. 2021 Apr 15;329:144-147. doi: 10.1016/j.ijcard.2020.12.044.
Excluded (reason: outside the field of interest)

77: Ozga M, Zhao Q, Benson D Jr, Elder P, Williams N, Bumma N, Rosko A, Chaudhry M, Khan A, Devarakonda S, Kahwash R, Vallakati A, Campbell C, Parikh SV, Almaani S, Prosek J, Bittengle J, Pfund K, LoRusso S, Freimer M, Redder E, Efebera Y, Sharma N. AL amyloidosis: The effect of fluorescent in situ hybridization abnormalities on organ involvement and survival. Cancer Med. 2021 Feb;10(3):965-973. doi: 10.1002/cam4.3683.
Excluded (reason: outside the field of interest)

78: Bonderman D, Pölzl G, Ablasser K, Agis H, Aschauer S, Auer-Grumbach M, Binder C, Dörler J, Duca F, Ebner C, Hacker M, Kain R, Kammerlander A, Koschutnik M, Kroiss AS, Mayr A, Nitsche C, Rainer PP, Reiter-Malmqvist S, Schneider M, Schwarz R, Verheyen N, Weber T, Zaruba MM, Badr Eslam R, Hülsmann M, Mascherbauer J. Diagnosis and treatment of cardiac amyloidosis: an interdisciplinary consensus statement. Wien Klin Wochenschr. 2020 Dec;132(23-24):742-761. doi: 10.1007/s00508-020-01781-z.
Excluded (reason: outside the field of interest)

79: Lindmark K, Pilebro B, Sundström T, Lindqvist P. Prevalence of wild type transtyrethin cardiac amyloidosis in a heart failure clinic. ESC Heart Fail. 2021 Feb;8(1):745-749. doi: 10.1002/ehf2.13110.
Excluded (reason: outside the field of interest)

80: Nitsche C, Scully PR, Patel KP, Kammerlander AA, Koschutnik M, Dona C, Wollenweber T, Ahmed N, Thornton GD, Kelion AD, Sabharwal N, Newton JD, Ozkor M, Kennon S, Mullen M, Lloyd G, Fontana M, Hawkins PN, Pugliese F, Menezes LJ, Moon JC, Mascherbauer J, Treibel TA. Prevalence and Outcomes of Concomitant Aortic Stenosis and Cardiac Amyloidosis. J Am Coll Cardiol. 2021 Jan 19;77(2):128-139. doi: 10.1016/j.jacc.2020.11.006.
Excluded (reason: outside the field of interest)

81: Ono R, Takahashi H, Sato T, Yamazaki T, Hori Y, Fukushima K. A case of cardiac amyloidosis in an elderly Japanese patient with amyloidogenic transthyretin Val122Ile variant. J Cardiol Cases. 2020 Jul 29;22(5):221-225. doi: 10.1016/j.jccase.2020.07.010.
Excluded (reason: outside the field of interest)

82: Lee Chuy K, Drill E, Yang JC, Landau H, Hassoun H, Nahhas O, Chen CL, Yu AF, Steingart RM, Liu JE. Incremental Value of Global Longitudinal Strain for Predicting Survival in Patients With Advanced AL Amyloidosis. JACC CardioOncol. 2020 Jun;2(2):223-231. doi: 10.1016/j.jaccao.2020.05.012.
Excluded (reason: outside the field of interest)

83: Suomalainen O, Pilv J, Loimaala A, Mätzke S, Heliö T, Uusitalo V. Prognostic significance of incidental suspected transthyretin amyloidosis on routine bone scintigraphy. J Nucl Cardiol. 2022 Jun;29(3):1021-1029. doi: 10.1007/s12350-020-02396-7.
Included in the systematic review and meta-analysis

84: Porcari A, Merlo M, Rapezzi C, Sinagra G. Transthyretin amyloid cardiomyopathy: An uncharted territory awaiting discovery. Eur J Intern Med. 2020 Dec;82:7-15. doi: 10.1016/j.ejim.2020.09.025.
Excluded (reason: outside the field of interest)

85: Gawor M, Holcman K, Franaszczyk M, Lipowska M, Michałek P, Teresińska A, Bilińska ZT, Rubiś P, Kostkiewicz M, Szot W, Podolec P, Grzybowski J. Spectrum of transthyretin gene mutations and clinical characteristics of Polish patients with cardiac transthyretin amyloidosis. Cardiol J. 2022;29(6):985-993. doi: 10.5603/CJ.a2020.0104.
Excluded (reason: outside the field of interest)

86: Grosse C, Grosse A, Salzer HJF, Dünser MW, Motz R, Langer R. Analysis of cardiopulmonary findings in COVID-19 fatalities: High incidence of pulmonary artery thrombi and acute suppurative bronchopneumonia. Cardiovasc Pathol. 2020 Nov-Dec;49:107263. doi: 10.1016/j.carpath.2020.107263.
Excluded (reason: outside the field of interest)

87: Ak Sivrikoz I, Çavuşoğlu Y. Transtiretin kardiyak amiloidozda kardiyak sintigrafi odaklı tanısal süreç [Cardiac scintigraphy-centered diagnostic process in transthyretin cardiac amyloidosis]. Turk Kardiyol Dern Ars. 2020 Jul;48(5):514-521. Turkish. doi: 10.5543/tkda.2020.90914.
Excluded (reason: outside the field of interest)

88: Porcari A, Falco L, Lio V, Merlo M, Fabris E, Bussani R, Sinagra G. Cardiac amyloidosis: do not forget to look for it. Eur Heart J Suppl. 2020 Jun;22(Suppl E):E142-E147. doi: 10.1093/eurheartj/suaa080.
Excluded (reason: outside the field of interest)

89: de Haro-Del Moral FJ, Pubul-Núñez V, Casáns-Tormo I, Jiménez-Heffernan A, Ruano-Pérez R. Cardiac scintigraphy with ^{99m}Tc-diphosphonates in cardiac amyloidosis. Rev Esp Med Nucl Imagen Mol (Engl Ed). 2020 Jul-Aug;39(4):254-266. English, Spanish. doi: 10.1016/j.remn.2020.03.007.
Excluded (reason: outside the field of interest)

90: Masri A, Bukhari S, Eisele YS, Soman P. Molecular Imaging of Cardiac Amyloidosis. J Nucl Med. 2020 Jul;61(7):965-970. doi: 10.2967/jnumed.120.245381.
Excluded (reason: outside the field of interest)

91: Gentile L, Di Bella G, Minutoli F, Cucinotta F, Obici L, Mussinelli R, Arimatea I, Russo M, Toscano A, Vita G, Mazzeo A. Description of a large cohort of Caucasian patients with V122I ATTRv amyloidosis: Neurological and cardiological features. J Peripher Nerv Syst. 2020 Sep;25(3):273-278. doi: 10.1111/jns.12385.
Excluded (reason: outside the field of interest)

92: Cuscaden C, Ramsay SC, Prasad S, Goodwin B, Smith J. Estimation of prevalence of transthyretin (ATTR) cardiac amyloidosis in an Australian subpopulation using bone scans with echocardiography and clinical correlation. J Nucl Cardiol. 2021 Dec;28(6):2845-2856. doi: 10.1007/s12350-020-02152-x.
Included in the systematic review and meta-analysis

93: Papathanasiou M, Carpinteiro A, Rischpler C, Hagenacker T, Rassaf T, Luedike P. Diagnosing cardiac amyloidosis in every-day practice: A practical guide for the cardiologist. Int J Cardiol Heart Vasc. 2020 Apr 27;28:100519. doi: 10.1016/j.ijcha.2020.100519.
Excluded (reason: outside the field of interest)

94: Fathala A. Incidentally detected cardiac amyloidosis on ^{99m}Tc-MDP bone scintigraphy. Radiol Case Rep. 2020 Apr 7;15(6):705-708. doi: 10.1016/j.radcr.2020.03.010.
Excluded (reason: case report)

95: Scully PR, Patel KP, Treibel TA, Thornton GD, Hughes RK, Chadalavada S, Katsoulis M, Hartman N, Fontana M, Pugliese F, Sabharwal N, Newton JD, Kelion A, Ozkor M, Kennon S, Mullen M, Lloyd G, Menezes LJ, Hawkins PN, Moon JC. Prevalence and outcome of dual aortic stenosis and cardiac amyloid pathology in patients referred for transcatheter aortic valve implantation. Eur Heart J. 2020 Aug 1;41(29):2759-2767. doi: 10.1093/eurheartj/ehaa170.
Excluded (reason: outside the field of interest)

96: Asif T, Araujo T, Singh V, Malhotra S. High prevalence of heart failure with reduced ejection fraction in patients with transthyretin cardiac amyloidosis. J Nucl Cardiol. 2020 Jun;27(3):1044-1046. doi: 10.1007/s12350-020-02098-0.
Excluded (reason: outside the field of interest)

97: Ikebe S, Takashio S, Nishi M, Morioka M, Tsujita K. Transthyretin Amyloid Cardiomyopathy Diagnosed on Incidental Myocardial Uptake During Bone Scintigraphy. Circ J. 2020 Mar 25;84(4):679. doi: 10.1253/circj.CJ-19-1155.
Excluded (reason: outside the field of interest)

98: Hansen PR, Krakauer M. [Transthyretin amyloid cardiomyopathy]. Ugeskr Laeger. 2020 Feb 3;182(6):V11190627.
Excluded (reason: outside the field of interest)

99: Nitsche C, Aschauer S, Kammerlander AA, Schneider M, Poschner T, Duca F, Binder C, Koschutnik M, Stiftinger J, Goliasch G, Siller-Matula J, Winter MP, Anvari-Pirsch A, Andreas M, Geppert A, Beitzke D, Loewe C, Hacker M, Agis H, Kain R, Lang I, Bonderman D, Hengstenberg C, Mascherbauer J. Light-chain and transthyretin cardiac amyloidosis in severe aortic stenosis: prevalence, screening possibilities, and outcome. Eur J Heart Fail. 2020 Oct;22(10):1852-1862. doi: 10.1002/ejhf.1756.
Excluded (reason: outside the field of interest)

100: Mircsof D. Diagnosis of Amyloidosis: A Survey of Current Awareness and Clinical Challenges Among Cardiologists in Switzerland. Cardiol Ther. 2020 Jun;9(1):127-138. doi: 10.1007/s40119-019-00160-8.
Excluded (reason: outside the field of interest)

101: Griffin JM, Maurer MS. Transthyretin cardiac amyloidosis: A treatable form of heart failure with a preserved ejection fraction. Trends Cardiovasc Med. 2021 Jan;31(1):59-66. doi: 10.1016/j.tcm.2019.12.003.
Excluded (reason: outside the field of interest)

102: Sidana S, Larson DP, Greipp PT, He R, McPhail ED, Dispenzieri A, Murray DL, Dasari S, Ansell SM, Muchtar E, Gonsalves WI, Kourelis TV, Ramirez-Alvarado M, Kapoor P, Rajkumar SV, Lacy MQ, Buadi FK, Leung N, Kyle RA, Kumar SK, King RL, Gertz MA. IgM AL amyloidosis: delineating disease biology and outcomes with clinical, genomic and bone marrow morphological features. Leukemia. 2020 May;34(5):1373-1382. doi: 10.1038/s41375-019-0667-6.

Excluded (reason: outside the field of interest)

103: Muchtar E, Gertz MA, Kourelis TV, Sidana S, Go RS, Lacy MQ, Buadi FK, Dingli D, Hayman SR, Kapoor P, Leung N, Fonder A, Hobbs M, Lisa Hwa Y, Gonsalves W, Warsame R, Russell S, Lust JA, Lin Y, Zeldenrust S, Rajkumar SV, Kyle RA, Kumar SK, Dispenzieri A. Bone marrow plasma cells 20% or greater discriminate presentation, response, and survival in AL amyloidosis. Leukemia. 2020 Apr;34(4):1135-1143. doi: 10.1038/s41375-019-0655-x.
Excluded (reason: outside the field of interest)

104: Lo Presti S, Horvath SA, Mihos CG, Rajadhyaksha C, McCloskey V, Santana O. Transthyretin Cardiac Amyloidosis as Diagnosed by 99mTc-PYP Scanning in Patients with Acute Heart Failure and Preserved Ejection Fraction. Crit Pathw Cardiol. 2019 Dec;18(4):195-199. doi: 10.1097/HPC.0000000000000183.
Excluded (reason: outside the field of interest)

105: Giannini G, Nast CC. An Organ System-Based Approach to Differential Diagnosis of Amyloid Type in Surgical Pathology. Arch Pathol Lab Med. 2020 Mar;144(3):379-387. doi: 10.5858/arpa.2018-0509-RA.
Excluded (reason: outside the field of interest)

106: Lopes LR, Futema M, Akhtar MM, Lorenzini M, Pittman A, Syrris P, Elliott PM. Prevalence of <i>TTR</i> variants detected by whole-exome sequencing in hypertrophic cardiomyopathy. Amyloid. 2019 Dec;26(4):243-247. doi: 10.1080/13506129.2019.1665996.
Excluded (reason: outside the field of interest)

107: Schwotzer R, Manz MG, Pederiva S, Waibel C, Caspar C, Lerch E, Flammer AJ, Brouwers S, Seeger H, Heimgartner R, Fehr T, Rossi D, Bianchi E, Stüssi G, Ghilardi G, Gerber B. Daratumumab for relapsed or refractory AL amyloidosis with high plasma cell burden. Hematol Oncol. 2019 Dec;37(5):595-600. doi: 10.1002/hon.2677.
Excluded (reason: outside the field of interest)

108: Di Giovanni B, Gustafson D, Delgado DH. Amyloid transthyretin cardiac amyloidosis: diagnosis and management. Expert Rev Cardiovasc Ther. 2019 Sep;17(9):673-681. doi: 10.1080/14779072.2019.1662723.
Excluded (reason: outside the field of interest)

109: Maurizi N, Rella V, Fumagalli C, Salerno S, Castelletti S, Dagradi F, Torchio M, Marceca A, Meda M, Gasparini M, Boschi B, Girolami F, Parati G, Olivotto I, Crotti L, Cecchi F. Prevalence of cardiac amyloidosis among adult patients referred to tertiary centres with an initial diagnosis of hypertrophic cardiomyopathy. Int J Cardiol. 2020 Feb 1;300:191-195. doi: 10.1016/j.ijcard.2019.07.051.
Excluded (reason: outside the field of interest)

110: López-Sainz Á, de Haro-Del Moral FJ, Dominguez F, Restrepo-Cordoba A, Amor-Salamanca A, Hernandez-Hernandez A, Ruiz-Guerrero L, Krsnik I, Cobo-Marcos M, Castro V, Toquero-Ramos J, Lara-Pezzi E, Fernandez-Lozano I, Alonso-Pulpon L, González-López E, Garcia-Pavia P. Prevalence of cardiac amyloidosis among elderly patients with systolic heart failure or conduction disorders. Amyloid. 2019 Sep;26(3):156-163. doi: 10.1080/13506129.2019.1625322.
Excluded (reason: outside the field of interest)

111: Tsutsui Y, Kubota T, Kato S, Nozoe M, Suematsu N, Okabe M, Yamamoto Y, Tsutsui H. Utility of ^{99 m}Tc-Pyrophosphate Scintigraphy in Diagnosing Transthyretin Cardiac Amyloidosis in Real-World Practice. Circ Rep. 2019 Jun 14;1(7):277-285. doi: 10.1253/circrep.CR-19-0015.
Excluded (reason: outside the field of interest)

112: Marume K, Takashio S, Nishi M, Hirakawa K, Yamamoto M, Hanatani S, Oda S, Utsunomiya D, Shiraishi S, Ueda M, Yamashita T, Sakamoto K, Yamamoto E, Kaikita K, Izumiya Y, Yamashita Y, Ando Y, Tsujita K. Combination of Commonly Examined Parameters Is a Useful Predictor of Positive ^{99 m}Tc-Labeled

Pyrophosphate Scintigraphy Findings in Elderly Patients With Suspected Transthyretin Cardiac Amyloidosis. Circ J. 2019 Jul 25;83(8):1698-1708. doi: 10.1253/circj.CJ-19-0255.
Excluded (reason: outside the field of interest)

113: Trachtenberg BH, Kamble RT, Rice L, Araujo-Gutierrez R, Bhimaraj A, Guha A, Park MH, Hussain I, Bruckner BA, Suarez EE, Victor DW, Adrogue HE, Baker KR, Estep JD. Delayed autologous stem cell transplantation following cardiac transplantation experience in patients with cardiac amyloidosis. Am J Transplant. 2019 Oct;19(10):2900-2909. doi: 10.1111/ajt.15487.
Excluded (reason: outside the field of interest)

114: Hugelshofer S, N'koulou R, Monney P. Amyloïdose cardiaque: une maladie rare ? [Cardiac amyloidosis : a rare disease ?]. Rev Med Suisse. 2019 May 22;15(652):1054-1059.
Excluded (reason: outside the field of interest)

115: Dang J, Abulizi M, Moktefi A, El Karoui K, Deux JF, Bodez D, Le Bras F, Belhadj K, Remy P, Issaurat P, Plante-Bordeneuve V, Molinier-Frenkel V, Fanen P, Guendouz S, Kharoubi M, Itti E, Damy T, Audard V. Renal Infarction and Its Consequences for Renal Function in Patients With Cardiac Amyloidosis. Mayo Clin Proc. 2019 Jun;94(6):961-975. doi: 10.1016/j.mayocp.2019.02.012.
Excluded (reason: outside the field of interest)

116: Kim HM, Sohn DW, Paeng JC. Prevalence of Positive ^{99 m}Tc-DPD Scintigraphy as an Indicator of the Prevalence of Wild-type Transthyretin Amyloidosis in the Elderly. Int Heart J. 2019 May 30;60(3):643-647. doi: 10.1536/ihj.18-345.
Included in the systematic review and meta-analysis

117: Sidana S, Tandon N, Gertz MA, Dispenzieri A, Ramirez-Alvarado M, Murray DL, Kourelis TV, Buadi FK, Kapoor P, Gonsalves W, Warsame R, Lacy MQ, Kyle RA, Rajkumar SV, Kumar SK, Leung N. Clinical features, laboratory characteristics and outcomes of patients with renal versus cardiac light chain amyloidosis. Br J Haematol. 2019 May;185(4):701-707. doi: 10.1111/bjh.15832.
Excluded (reason: outside the field of interest)

118: Feng J, Zhang C, Shen K, Sun J, Fang Q, Zhang L, Cao X, Zhou D, Li J, Tian Z. Outcome of Cardiac Light-Chain Amyloidosis in the Era of Novel Therapy – A Single-Center Cohort Study of 227 Patients. Circ J. 2019 Mar 25;83(4):775-782. doi: 10.1253/circj.CJ-18-1048.
Excluded (reason: outside the field of interest)

119: Ramaekers J, Janssens J, Waumans L, Stessens L, Dupont M, Mullens W, Martens P. Indications and diagnostic yield of endomyocardial biopsies for unexplained cardiomyopathy, a single center experience. Acta Cardiol. 2020 Apr;75(2):138-146. doi: 10.1080/00015385.2018.1561597.
Excluded (reason: outside the field of interest)

120: Fukui T, Tanimura Y, Matsumoto Y, Horitani S, Tomiyama T, Okazaki K. Incidentally Detected Amyloid Light-Chain Amyloidosis Caused by Monoclonal Gammopathy of Undetermined Significance: Possible Time-Dependent Change in Colonic Findings. Case Rep Gastroenterol. 2018 Dec 6;12(3):737-746. doi: 10.1159/000494919.
Excluded (reason: outside the field of interest)

121: Sperry BW, Reyes BA, Ikram A, Donnelly JP, Phelan D, Jaber WA, Shapiro D, Evans PJ, Maschke S, Kilpatrick SE, Tan CD, Rodriguez ER, Monteiro C, Tang WHW, Kelly JW, Seitz WH Jr, Hanna M. Tenosynovial and Cardiac Amyloidosis in Patients Undergoing Carpal Tunnel Release. J Am Coll Cardiol. 2018 Oct 23;72(17):2040-2050. doi: 10.1016/j.jacc.2018.07.092.
Excluded (reason: outside the field of interest)

122: Povar Echeverría M, Auquilla Clavijo PE, Escobedo Palau JA, Navarro Beltrán P, Povar Marco J. Diagnóstico no invasivo de amiloidosis cardiaca por transtirretina. Caso clínico [Non-invasive diagnosis of cardiac amyloidosis due to transthyretin. Case report]. An Sist Sanit Navar. 2018 Aug 29;41(2):263-267. Spanish. doi: 10.23938/ASSN.0295.
Excluded (reason: outside the field of interest)

123: Mohamed-Salem L, Santos-Mateo JJ, Sanchez-Serna J, Hernández-Vicente Á, Reyes-Marle R, Castellón Sánchez MI, Claver-Valderas MA, Gonzalez-Vioque E, Haro-Del Moral FJ, García-Pavía P, Pascual-Figal DA. Prevalence of wild type ATTR assessed as myocardial uptake in bone scan in the elderly population. Int J Cardiol. 2018 Nov 1;270:192-196. doi: 10.1016/j.ijcard.2018.06.006.
Included in the systematic review and meta-analysis

124: Sidiqi MH, Aljama MA, Jevremovic D, Morice WG, Timm M, Buadi FK, Warsame R, Lacy MQ, Dispenzieri A, Dingli D, Gonsalves WI, Kumar S, Kapoor P, Kourelis T, Leung N, Hogan WJ, Gertz M. Plasma cell proliferative index predicts outcome in immunoglobulin light chain amyloidosis treated with stem cell transplantation. Haematologica. 2018 Jul;103(7):1229-1234. doi: 10.3324/haematol.2018.189985.
Excluded (reason: outside the field of interest)

125: Martinez-Naharro A, Kotecha T, Norrington K, Boldrini M, Rezk T, Quarta C, Treibel TA, Whelan CJ, Knight DS, Kellman P, Ruberg FL, Gillmore JD, Moon JC, Hawkins PN, Fontana M. Native T1 and Extracellular Volume in Transthyretin Amyloidosis. JACC Cardiovasc Imaging. 2019 May;12(5):810-819. doi: 10.1016/j.jcmg.2018.02.006.
Excluded (reason: outside the field of interest)

126: Tovar N, Rodríguez-Lobato LG, Cibeira MT, Magnano L, Isola I, Rosiñol L, Bladé J, Fernández de Larrea C. Bone marrow plasma cell infiltration in light chain amyloidosis: impact on organ involvement and outcome. Amyloid. 2018 Jun;25(2):79-85. doi: 10.1080/13506129.2018.1443439.
Excluded (reason: outside the field of interest)

127: Ng QKT, Sethi P, Saunders TA, Pampaloni MH, Flavell RR. Discordant Findings on 18F-NaF and 99mTc-HDP Bone Scans in a Patient With ATTR Cardiac Amyloidosis. Clin Nucl Med. 2018 Mar;43(3):e89-e92. doi: 10.1097/RLU.0000000000001933.
Excluded (reason: outside the field of interest)

128: Plante-Bordeneuve V. Transthyretin familial amyloid polyneuropathy: an update. J Neurol. 2018 Apr;265(4):976-983. doi: 10.1007/s00415-017-8708-4.
Excluded (reason: outside the field of interest)

129: Castaño A, Narotsky DL, Hamid N, Khalique OK, Morgenstern R, DeLuca A, Rubin J, Chiuzan C, Nazif T, Vahl T, George I, Kodali S, Leon MB, Hahn R, Bokhari S, Maurer MS. Unveiling transthyretin cardiac amyloidosis and its predictors among elderly patients with severe aortic stenosis undergoing transcatheter aortic valve replacement. Eur Heart J. 2017 Oct 7;38(38):2879-2887. doi: 10.1093/eurheartj/ehx350.
Excluded (reason: outside the field of interest)

130: Sperry BW, Vranian MN, Tower-Rader A, Hachamovitch R, Hanna M, Brunken R, Phelan D, Cerqueira MD, Jaber WA. Regional Variation in Technetium Pyrophosphate Uptake in Transthyretin Cardiac Amyloidosis and Impact on Mortality. JACC Cardiovasc Imaging. 2018 Feb;11(2 Pt 1):234-242. doi: 10.1016/j.jcmg.2017.06.020.
Excluded (reason: outside the field of interest)

131: Hammons L, Brazauskas R, Pasquini M, Hamadani M, Hari P, D'Souza A. Presence of fluorescent in situ hybridization abnormalities is associated with plasma cell burden in light chain amyloidosis. Hematol Oncol Stem Cell Ther. 2018 Jun;11(2):105-111. doi: 10.1016/j.hemonc.2017.07.005.
Excluded (reason: outside the field of interest)

132: Korkmaz C, Cansu DÜ, Sayer JA. Urolithiasis as an extraarticular manifestation of ankylosing spondylitis. Rheumatol Int. 2017 Dec;37(12):1949-1956. doi: 10.1007/s00296-017-3788-0.
Excluded (reason: outside the field of interest)

133: Dittrich T, Bochtler T, Kimmich C, Becker N, Jauch A, Goldschmidt H, Ho AD, Hegenbart U, Schönland SO. AL amyloidosis patients with low amyloidogenic free light chain levels at first diagnosis have an excellent prognosis. Blood. 2017 Aug 3;130(5):632-642. doi: 10.1182/blood-2017-02-767475.
Excluded (reason: outside the field of interest)

134: González-López E, Gagliardi C, Dominguez F, Quarta CC, de Haro-Del Moral FJ, Milandri A, Salas C, Cinelli M, Cobo-Marcos M, Lorenzini M, Lara-Pezzi E, Foffi S, Alonso-Pulpon L, Rapezzi C, Garcia-Pavia P. Clinical characteristics of wild-type transthyretin cardiac amyloidosis: disproving myths. Eur Heart J. 2017 Jun 21;38(24):1895-1904. doi: 10.1093/eurheartj/ehx043.
Excluded (reason: outside the field of interest)

135: Hutt DF, Fontana M, Burniston M, Quigley AM, Petrie A, Ross JC, Page J, Martinez-Naharro A, Wechalekar AD, Lachmann HJ, Quarta CC, Rezk T, Mahmood S, Sachchithanantham S, Youngstein T, Whelan CJ, Lane T, Gilbertson JA, Rowczenio D, Hawkins PN, Gillmore JD. Prognostic utility of the Perugini grading of 99mTc-DPD scintigraphy in transthyretin (ATTR) amyloidosis and its relationship with skeletal muscle and soft tissue amyloid. Eur Heart J Cardiovasc Imaging. 2017 Dec 1;18(12):1344-1350. doi: 10.1093/ehjci/jew325.
Excluded (reason: outside the field of interest)

136: Vranian MN, Sperry BW, Hanna M, Hachamovitch R, Ikram A, Brunken RC, Jaber WA. Technetium pyrophosphate uptake in transthyretin cardiac amyloidosis: Associations with echocardiographic disease severity and outcomes. J Nucl Cardiol. 2018 Aug;25(4):1247-1256. doi: 10.1007/s12350-016-0768-9.
Excluded (reason: outside the field of interest)

137: Fuijkschot WW, Morrison MC, van der Linden R, Krijnen PAJ, Zethof IPA, Theyse LFH, Kleemann R, Niessen HWM, Smulders YM. Orthopedic surgery increases atherosclerotic lesions and necrotic core area in ApoE-/- mice. Atherosclerosis. 2016 Dec;255:164-170. doi: 10.1016/j.atherosclerosis.2016.07.909.
Excluded (reason: outside the field of interest)

138: Bennani Smires Y, Victor G, Ribes D, Berry M, Cognet T, Méjean S, Huart A, Roussel M, Petermann A, Roncalli J, Carrié D, Rousseau H, Berry I, Chauveau D, Galinier M, Lairez O. Pilot study for left ventricular imaging phenotype of patients over 65 years old with heart failure and preserved ejection fraction: the high prevalence of amyloid cardiomyopathy. Int J Cardiovasc Imaging. 2016 Sep;32(9):1403-1413. doi: 10.1007/s10554-016-0915-z.
Excluded (reason: outside the field of interest)

139: Muchtar E, Derudas D, Mauermann M, Liewluck T, Dispenzieri A, Kumar SK, Dingli D, Lacy MQ, Buadi FK, Hayman SR, Kapoor P, Leung N, Chakraborty R, Gonsalves W, Russell S, Lust JA, Lin Y, Go RS, Zeldenrust S, Kyle RA, Rajkumar SV, Gertz MA. Systemic Immunoglobulin Light Chain Amyloidosis-Associated Myopathy: Presentation, Diagnostic Pitfalls, and Outcome. Mayo Clin Proc. 2016 Oct;91(10):1354-1361. doi: 10.1016/j.mayocp.2016.06.027.
Excluded (reason: outside the field of interest)

140: Rosengren S, Mellqvist UH, Nahi H, Forsberg K, Lenhoff S, Strömberg O, Ahlberg L, Linder O, Carlson K. Outcome of AL amyloidosis after high-dose melphalan and autologous stem cell transplantation in Sweden, long-term results from all patients treated in 1994-2009. Bone Marrow Transplant. 2016 Dec;51(12):1569-1572. doi: 10.1038/bmt.2016.249.
Excluded (reason: outside the field of interest)

141: Castano A, Haq M, Narotsky DL, Goldsmith J, Weinberg RL, Morgenstern R, Pozniakoff T, Ruberg FL, Miller EJ, Berk JL, Dispenzieri A, Grogan M, Johnson G, Bokhari S, Maurer MS. Multicenter Study of Planar Technetium

99m Pyrophosphate Cardiac Imaging: Predicting Survival for Patients With ATTR Cardiac Amyloidosis. JAMA Cardiol. 2016 Nov 1;1(8):880-889. doi: 10.1001/jamacardio.2016.2839.
Excluded (reason: outside the field of interest)

142: Nienhuis HL, Bijzet J, Hazenberg BP. The Prevalence and Management of Systemic Amyloidosis in Western Countries. Kidney Dis (Basel). 2016 Apr;2(1):10-9. doi: 10.1159/000444206.
Excluded (reason: outside the field of interest)

143: Treibel TA, Fontana M, Gilbertson JA, Castelletti S, White SK, Scully PR, Roberts N, Hutt DF, Rowczenio DM, Whelan CJ, Ashworth MA, Gillmore JD, Hawkins PN, Moon JC. Occult Transthyretin Cardiac Amyloid in Severe Calcific Aortic Stenosis: Prevalence and Prognosis in Patients Undergoing Surgical Aortic Valve Replacement. Circ Cardiovasc Imaging. 2016 Aug;9(8):e005066. doi: 10.1161/CIRCIMAGING.116.005066.
Excluded (reason: outside the field of interest)

144: Shay JW, Homma N, Zhou R, Naseer MI, Chaudhary AG, Al-Qahtani M, et al. Abstracts from the 3rd International Genomic Medicine Conference (3rd IGMC 2015): Jeddah, Kingdom of Saudi Arabia. 30 November - 3 December 2015. BMC Genomics. 2016 Jul 20;17 Suppl 6(Suppl 6):487. doi: 10.1186/s12864-016-2858-0.
Excluded (reason: outside the field of interest)

145: Harb SC, Haq M, Flood K, Guerrieri A, Passerell W, Jaber WA, Miller EJ. National patterns in imaging utilization for diagnosis of cardiac amyloidosis: A focus on Tc99m-pyrophosphate scintigraphy. J Nucl Cardiol. 2017 Jun;24(3):1094-1097. doi: 10.1007/s12350-016-0478-3.
Excluded (reason: outside the field of interest)

146: Wechalekar AD, Gillmore JD, Hawkins PN. Systemic amyloidosis. Lancet. 2016 Jun 25;387(10038):2641-2654. doi: 10.1016/S0140-6736(15)01274-X.
Excluded (reason: outside the field of interest)

147: Visconti L, Cernaro V, Ferrara D, Lacava V, Ricciardi CA, Di Mauro E, Amico L, Santoro D, Buemi M. Porpora periorbitaria: un segnale patognomonico ma tardivo di amiloidosi AL [Periorbital purpura: a pathognomonic but late sign of AL amyloidosis]. G Ital Nefrol. 2015 Sep-Oct;32(5):gin/32.5.8. Italian.
Excluded (reason: outside the field of interest)

148: González-López E, Gallego-Delgado M, Guzzo-Merello G, de Haro-Del Moral FJ, Cobo-Marcos M, Robles C, Bornstein B, Salas C, Lara-Pezzi E, Alonso-Pulpon L, Garcia-Pavia P. Wild-type transthyretin amyloidosis as a cause of heart failure with preserved ejection fraction. Eur Heart J. 2015 Oct 7;36(38):2585-94. doi: 10.1093/eurheartj/ehv338.
Excluded (reason: outside the field of interest)

149: Kastritis E, Gavriatopoulou M, Dimopoulos MA, Eleutherakis-Papaiakovou E, Kanellias N, Roussou M, Pamboucas C, Toumanidis ST, Terpos E. Osteoprotegerin is a significant prognostic factor for overall survival in patients with primary systemic amyloidosis independent of the Mayo staging. Blood Cancer J. 2015 Jun 5;5(6):e319. doi: 10.1038/bcj.2015.45.
Excluded (reason: outside the field of interest)

150: Warsame R, Kumar SK, Gertz MA, Lacy MQ, Buadi FK, Hayman SR, Leung N, Dingli D, Lust JA, Ketterling RP, Lin Y, Russell S, Hwa L, Kapoor P, Go RS, Zeldenrust SR, Kyle RA, Rajkumar SV, Dispenzieri A. Abnormal FISH in patients with immunoglobulin light chain amyloidosis is a risk factor for cardiac involvement and for death. Blood Cancer J. 2015 May 1;5(5):e310. doi: 10.1038/bcj.2015.34.
Excluded (reason: outside the field of interest)

151: Cornell RF, Zhong X, Arce-Lara C, Atallah E, Blust L, Drobyski WR, Fenske TS, Pasquini MC, Rizzo JD, Saber W, Hari PN. Bortezomib-based induction for transplant ineligible AL amyloidosis and feasibility of later transplantation. Bone Marrow Transplant. 2015 Jul;50(7):914-7. doi: 10.1038/bmt.2015.73.
Excluded (reason: outside the field of interest)

152: Bohlega S, Monies DM, Abulaban AA, Murad HN, Alhindi HN, Meyer BF. Clinical and genetic features of anoctaminopathy in Saudi Arabia. Neurosciences (Riyadh). 2015 Apr;20(2):173-7. doi: 10.17712/nsj.2015.2.20140547.
Excluded (reason: outside the field of interest)

153: Kongtim P, Qazilbash MH, Shah JJ, Hamdi A, Shah N, Bashir Q, Wang M, Champlin R, Manasanch EE, Weber D, Orlowski RZ, Parmar S. High-dose therapy with auto-SCT is feasible in high-risk cardiac amyloidosis. Bone Marrow Transplant. 2015 May;50(5):668-72. doi: 10.1038/bmt.2015.21.
Excluded (reason: outside the field of interest)

154: Nelson LM, Gustafsson F, Gimsing P. Characteristics and long-term outcome of patients with systemic immunoglobulin light-chain amyloidosis. Acta Haematol. 2015;133(4):336-46. doi: 10.1159/000363682.
Excluded (reason: outside the field of interest)

155: Wachsmann JW, Gerbaudo VH. Thorax: normal and benign pathologic patterns in FDG-PET/CT imaging. PET Clin. 2014 Apr;9(2):147-68. doi: 10.1016/j.cpet.2013.10.004.
Excluded (reason: outside the field of interest)

156: Demirel K, Sadic M, Korkmaz M, Comak A, Atilgan HI, Koca G. Diffuse Myocardial Uptake of (99m)Tc-HDP in Multiple Myeloma. Nucl Med Mol Imaging. 2013 Sep;47(3):208-11. doi: 10.1007/s13139-013-0209-5.
Excluded (reason: outside the field of interest)

157: Longhi S, Guidalotti PL, Quarta CC, Gagliardi C, Milandri A, Lorenzini M, Potena L, Leone O, Bartolomei I, Pastorelli F, Salvi F, Rapezzi C. Identification of TTR-related subclinical amyloidosis with 99mTc-DPD scintigraphy. JACC Cardiovasc Imaging. 2014 May;7(5):531-2. doi: 10.1016/j.jcmg.2014.03.004.
Included in the systematic review and meta-analysis

158: Arbustini E, Merlini G. Early identification of transthyretin-related hereditary cardiac amyloidosis. JACC Cardiovasc Imaging. 2014 May;7(5):511-4. doi: 10.1016/j.jcmg.2014.03.007.
Excluded (reason: outside the field of interest)

159: Kristen AV, Scherer K, Buss S, aus dem Siepen F, Haufe S, Bauer R, Hinderhofer K, Giannitsis E, Hardt S, Haberkorn U, Katus HA, Steen H. Noninvasive risk stratification of patients with transthyretin amyloidosis. JACC Cardiovasc Imaging. 2014 May;7(5):502-10. doi: 10.1016/j.jcmg.2014.03.002.
Excluded (reason: outside the field of interest)

160: Caobelli F, Paghera B, Pizzocaro C, Guerra UP. Extraosseous myocardial uptake incidentally detected during bone scan: report of three cases and a systematic literature review of extraosseous uptake. Nucl Med Rev Cent East Eur. 2013;16(2):82-7. doi: 10.5603/NMR.2013.0040.
Excluded (reason: small case series)

161: Hernández-Reyes J, Galo-Hooker E, Ruiz-Delgado GJ, Ruiz-Argüelles GJ. Systemic immunoglobulin light-chain amyloidosis (AL) in Mexico: a single institution, 30-year experience. Rev Invest Clin. 2012 Nov-Dec;64(6 Pt 2):604-8.
Excluded (reason: outside the field of interest)

162: Fitzgerald BT, Bashford J, Scalia GM. The return of the normal heart: resolution of cardiac amyloidosis after chemotherapy and bone marrow transplantation. Heart Lung Circ. 2013 Aug;22(8):655-60. doi: 10.1016/j.hlc.2013.01.013.

Excluded (reason: outside the field of interest)

163: Dinner S, Witteles W, Witteles R, Lam A, Arai S, Lafayette R, George TI, Schrier SL, Liedtke M. The prognostic value of diagnosing concurrent multiple myeloma in immunoglobulin light chain amyloidosis. Br J Haematol. 2013 May;161(3):367-72. doi: 10.1111/bjh.12269.
Excluded (reason: outside the field of interest)

164: Nasr SH, Said SM, Valeri AM, Sethi S, Fidler ME, Cornell LD, Gertz MA, Dispenzieri A, Buadi FK, Vrana JA, Theis JD, Dogan A, Leung N. The diagnosis and characteristics of renal heavy-chain and heavy/light-chain amyloidosis and their comparison with renal light-chain amyloidosis. Kidney Int. 2013 Mar;83(3):463-70. doi: 10.1038/ki.2012.414.
Excluded (reason: outside the field of interest)

165: Russo M, Mazzeo A, Stancanelli C, Di Leo R, Gentile L, Di Bella G, Minutoli F, Baldari S, Vita G. Transthyretin-related familial amyloidotic polyneuropathy: description of a cohort of patients with Leu64 mutation and late onset. J Peripher Nerv Syst. 2012 Dec;17(4):385-90. doi: 10.1111/j.1529-8027.2012.00436.x.
Excluded (reason: outside the field of interest)

166: Battaglia Y, Granata A, Zamboli P, Lusenti T, Di Lullo L, Floccari F, Logias F, D'Amelio A, Fiorini F. Il nefrologo e la gestione ecocolorDoppler del paziente in dialisi [Management of color-Doppler imaging in dialysis patients]. G Ital Nefrol. 2012 Nov-Dec;29(6):683-9. Italian.
Excluded (reason: outside the field of interest)

167: Gertz MA, Lacy MQ, Dispenzieri A, Kumar SK, Dingli D, Leung N, Hogan WJ, Buadi FK, Hayman SR. Refinement in patient selection to reduce treatment-related mortality from autologous stem cell transplantation in amyloidosis. Bone Marrow Transplant. 2013 Apr;48(4):557-61. doi: 10.1038/bmt.2012.170.
Excluded (reason: outside the field of interest)

168: Inamori Y, Higuchi I, Inoue T, Sakiyama Y, Hashiguchi A, Higashi K, Shiraishi T, Okubo R, Arimura K, Mitsuyama Y, Takashima H. Inclusion body myositis coexisting with hypertrophic cardiomyopathy: an autopsy study. Neuromuscul Disord. 2012 Aug;22(8):747-54. doi: 10.1016/j.nmd.2012.03.011.
Excluded (reason: outside the field of interest)

169: Dickens AP, Lang IA, Langa KM, Kos K, Llewellyn DJ. Vitamin D, cognitive dysfunction and dementia in older adults. CNS Drugs. 2011 Aug;25(8):629-39. doi: 10.2165/11593080-000000000-00000.
Excluded (reason: outside the field of interest)

170: Kristen AV, Haufe S, Schonland SO, Hegenbart U, Schnabel PA, Röcken C, Hardt S, Lohse P, Ho AD, Haberkorn U, Dengler TJ, Altland K, Katus HA. Skeletal scintigraphy indicates disease severity of cardiac involvement in patients with senile systemic amyloidosis. Int J Cardiol. 2013 Apr 5;164(2):179-84. doi: 10.1016/j.ijcard.2011.06.123.
Excluded (reason: outside the field of interest)

171: Rapezzi C, Quarta CC, Guidalotti PL, Pettinato C, Fanti S, Leone O, Ferlini A, Longhi S, Lorenzini M, Reggiani LB, Gagliardi C, Gallo P, Villani C, Salvi F. Role of (99m)Tc-DPD scintigraphy in diagnosis and prognosis of hereditary transthyretin-related cardiac amyloidosis. JACC Cardiovasc Imaging. 2011 Jun;4(6):659-70. doi: 10.1016/j.jcmg.2011.03.016.
Excluded (reason: outside the field of interest)

172: Gertz MA. Immunoglobulin light chain amyloidosis: 2011 update on diagnosis, risk-stratification, and management. Am J Hematol. 2011 Feb;86(2):180-6. doi: 10.1002/ajh.21934.
Excluded (reason: outside the field of interest)

173: Gertz MA, Lacy MQ, Dispenzieri A, Kumar SK, Buadi FK, Dingli D, Leung N, Hogan WJ, Hayman SR. Trends in day 100 and 2-year survival after auto-SCT for AL amyloidosis: outcomes before and after 2006. Bone Marrow Transplant. 2011 Jul;46(7):970-5. doi: 10.1038/bmt.2010.234.
Excluded (reason: outside the field of interest)

174: Koike H, Kiuchi T, Iijima M, Ueda M, Ando Y, Morozumi S, Tomita M, Kawagashira Y, Watanabe H, Katsuno M, Shimoyama Y, Okazaki Y, Kamei H, Sobue G. Systemic but asymptomatic transthyretin amyloidosis 8 years after domino liver transplantation. J Neurol Neurosurg Psychiatry. 2011 Nov;82(11):1287-90. doi: 10.1136/jnnp.2010.218958.
Excluded (reason: outside the field of interest)

175: Fujii T, Komatsu Y, Yasoda A, Kondo E, Yoshioka T, Nambu T, Kanamoto N, Miura M, Tamura N, Arai H, Mukoyama M, Nakao K. Circulating C-type natriuretic peptide (CNP) rescues chondrodysplastic CNP knockout mice from their impaired skeletal growth and early death. Endocrinology. 2010 Sep;151(9):4381-8. doi: 10.1210/en.2010-0078.
Excluded (reason: outside the field of interest)

176: Michael M, Kastritis E, Delimpassi S, Michalis E, Repoussis P, Kyrtsonis MC, Katodritou E, Anagnostopoulos N, Zervas K, Dimopoulos MA; Greek Myeloma Study Group. Clinical characteristics and outcome of primary systemic light-chain amyloidosis in Greece. Clin Lymphoma Myeloma Leuk. 2010 Feb;10(1):56-61. doi: 10.3816/CLML.2010.n.006.
Excluded (reason: outside the field of interest)

177: Madan S, Dispenzieri A, Lacy MQ, Buadi F, Hayman SR, Zeldenrust SR, Rajkumar SV, Gertz MA, Kumar SK. Clinical features and treatment response of light chain (AL) amyloidosis diagnosed in patients with previous diagnosis of multiple myeloma. Mayo Clin Proc. 2010 Mar;85(3):232-8. doi: 10.4065/mcp.2009.0547.
Excluded (reason: outside the field of interest)

178: Mignot A, Varnous S, Redonnet M, Jaccard A, Epailly E, Vermes E, Boissonnat P, Gandjbakhch I, Herpin D, Touchard G, Bridoux F. Heart transplantation in systemic (AL) amyloidosis: a retrospective study of eight French patients. Arch Cardiovasc Dis. 2008 Sep;101(9):523-32. doi: 10.1016/j.acvd.2008.06.018.
Excluded (reason: outside the field of interest)

179: Tazawa K, Matsuda M, Yoshida T, Gono T, Katoh N, Shimojima Y, Ishii W, Fushimi T, Koyama J, Ikeda S. Therapeutic outcome of cyclic VAD (vincristine, doxorubicin and dexamethasone) therapy in primary systemic AL amyloidosis patients. Intern Med. 2008;47(17):1517-22. doi: 10.2169/internalmedicine.47.0949.
Excluded (reason: outside the field of interest)

180: Wechalekar AD, Lachmann HJ, Goodman HJ, Bradwell A, Hawkins PN, Gillmore JD. AL amyloidosis associated with IgM paraproteinemia: clinical profile and treatment outcome. Blood. 2008 Nov 15;112(10):4009-16. doi: 10.1182/blood-2008-02-138156.
Excluded (reason: outside the field of interest)

181: Kruger DF. Exploring the pharmacotherapeutic options for treating type 2 diabetes. Diabetes Educ. 2008 May-Jun;34 Suppl 3:60S-65S. doi: 10.1177/0145721708319234.
Excluded (reason: outside the field of interest)

182: Tse KC, Lui SL, Lo WK. Comparison of long-term survival (beyond 12 years) in patients on peritoneal dialysis and on hemodialysis. Perit Dial Int. 2003 Dec;23 Suppl 2:S104-8.
Excluded (reason: outside the field of interest)

183: Wechalekar K, Ng FS, Poole-Wilson PA, Duncan A, Nutting C, Naidoo VV, Underwood SR. Cardiac amyloidosis diagnosed incidentally by bone scintigraphy. J Nucl Cardiol. 2007 Sep-Oct;14(5):750-3. doi: 10.1016/j.nuclcard.2007.07.002.
Excluded (reason: case report)

184: Tajima Y, Shimada A, Yamada TK, Cowan DF. Amyloidosis in two Stejneger's beaked whales (Mesoplodon stejnegeri) stranded at the Sea of Japan. J Zoo Wildl Med. 2007 Mar;38(1):108-13. doi: 10.1638/05-108.1.
Excluded (reason: outside the field of interest)

185: Jacobs P, Ruff P, Wood L, Moodley D, Mansvelt E. Amyloidosis: a changing clinical perspective. Hematology. 2007 Apr;12(2):163-7. doi: 10.1080/10245330701214350.
Excluded (reason: outside the field of interest)

186: Roy A, Roy V. Primary systemic amyloidosis. Early diagnosis and therapy can improve survival rates and quality of life. Postgrad Med. 2006 Jun- Jul;119(1):93-9. doi: 10.3810/pgm.2006.06.1652.
Excluded (reason: outside the field of interest)

187: Saito A, Gejyo F. Current clinical aspects of dialysis-related amyloidosis in chronic dialysis patients. Ther Apher Dial. 2006 Aug;10(4):316-20. doi: 10.1111/j.1744-9987.2006.00383.x.
Excluded (reason: outside the field of interest)

188: Vesole DH, Pérez WS, Akasheh M, Boudreau C, Reece DE, Bredeson CN; Plasma Cell Disorders Working Committee of the Center for International Blood and Marrow Transplant Research. High-dose therapy and autologous hematopoietic stem cell transplantation for patients with primary systemic amyloidosis: a Center for International Blood and Marrow Transplant Research Study. Mayo Clin Proc. 2006 Jul;81(7):880-8. doi: 10.4065/81.7.880.
Excluded (reason: outside the field of interest)

189: Butcovan D, Pintilie D, Prisecariu C, Dabija A, Ralea C, Mihailovici MS. Amiloidoza sistemică. Prezentare de caz [Systemic amyloidosis. Case report]. Rev Med Chir Soc Med Nat Iasi. 2005 Jul-Sep;109(3):507-10. Romanian.
Excluded (reason: outside the field of interest)

190: Gertz MA, Blood E, Vesole DH, Abonour R, Lazarus HM, Greipp PR. A multicenter phase 2 trial of stem cell transplantation for immunoglobulin light-chain amyloidosis (E4A97): an Eastern Cooperative Oncology Group Study. Bone Marrow Transplant. 2004 Jul;34(2):149-54. doi: 10.1038/sj.bmt.1704539.
Excluded (reason: outside the field of interest)

191: Dispenzieri A, Kyle RA, Lacy MQ, Therneau TM, Larson DR, Plevak MF, Rajkumar SV, Fonseca R, Greipp PR, Witzig TE, Lust JA, Zeldenrust SR, Snow DS, Hayman SR, Litzow MR, Gastineau DA, Tefferi A, Inwards DJ, Micallef IN, Ansell SM, Porrata LF, Elliott MA, Gertz MA. Superior survival in primary systemic amyloidosis patients undergoing peripheral blood stem cell transplantation: a case-control study. Blood. 2004 May 15;103(10):3960-3. doi: 10.1182/blood-2003-12-4192.
Excluded (reason: outside the field of interest)

192: Sanchorawala V, Wright DG, Seldin DC, Falk RH, Finn KT, Dember LM, Berk JL, Quillen K, Anderson JJ, Comenzo RL, Skinner M. High-dose intravenous melphalan and autologous stem cell transplantation as initial therapy or following two cycles of oral chemotherapy for the treatment of AL amyloidosis: results of a prospective randomized trial. Bone Marrow Transplant. 2004 Feb;33(4):381-8. doi: 10.1038/sj.bmt.1704346.
Excluded (reason: outside the field of interest)

193: Mollee PN, Wechalekar AD, Pereira DL, Franke N, Reece D, Chen C, Stewart AK. Autologous stem cell transplantation in primary systemic amyloidosis: the impact of selection criteria on outcome. Bone Marrow Transplant. 2004 Feb;33(3):271-7. doi: 10.1038/sj.bmt.1704344.

Excluded (reason: outside the field of interest)

194: Blum W, Khoury H, Lin HS, Vij R, Goodnough LT, Devine S, Dipersio J, Adkins D. Primary amyloidosis patients with significant organ dysfunction tolerate autologous transplantation after conditioning with single-dose total body irradiation alone: a feasibility study. Biol Blood Marrow Transplant. 2003 Jun;9(6):397-404. doi: 10.1016/s1083-8791(03)00106-x.
Excluded (reason: outside the field of interest)

195: Gertz MA, Kyle RA. Amyloidosis with IgM monoclonal gammopathies. Semin Oncol. 2003 Apr;30(2):325-8. doi: 10.1053/sonc.2003.50060.
Excluded (reason: outside the field of interest)

196: Hara S. [Dialysis therapy--initiation and major complications]. Nihon Jinzo Gakkai Shi. 2003;45(2):65-75. Japanese.
Excluded (reason: outside the field of interest)

197: Braun J, Pincus T. Mortality, course of disease and prognosis of patients with ankylosing spondylitis. Clin Exp Rheumatol. 2002 Nov-Dec;20(6 Suppl 28):S16-22.
Excluded (reason: outside the field of interest)

198: Turturro A, Duffy P, Hass B, Kodell R, Hart R. Survival characteristics and age-adjusted disease incidences in C57BL/6 mice fed a commonly used cereal-based diet modulated by dietary restriction. J Gerontol A Biol Sci Med Sci. 2002 Nov;57(11):B379-89. doi: 10.1093/gerona/57.11.b379.
Excluded (reason: outside the field of interest)

199: Pardanani A, Witzig TE, Schroeder G, McElroy EA, Fonseca R, Dispenzieri A, Lacy MQ, Lust JA, Kyle RA, Greipp PR, Gertz MA, Rajkumar SV. Circulating peripheral blood plasma cells as a prognostic indicator in patients with primary systemic amyloidosis. Blood. 2003 Feb 1;101(3):827-30. doi: 10.1182/blood-2002-06-1698.
Excluded (reason: outside the field of interest)

200: Sanchorawala V, Wright DG, Seldin DC, Dember LM, Finn K, Falk RH, Berk J, Quillen K, Skinner M. An overview of the use of high-dose melphalan with autologous stem cell transplantation for the treatment of AL amyloidosis. Bone Marrow Transplant. 2001 Oct;28(7):637-42. doi: 10.1038/sj.bmt.1703200.
Excluded (reason: outside the field of interest)

201: Gertz MA, Lacy MQ, Gastineau DA, Inwards DJ, Chen MG, Tefferi A, Kyle RA, Litzow MR. Blood stem cell transplantation as therapy for primary systemic amyloidosis (AL). Bone Marrow Transplant. 2000 Nov;26(9):963-9. doi: 10.1038/sj.bmt.1702643.
Excluded (reason: outside the field of interest)

202: Rives S, Bladé J, Martínez C, Marín P, Carreras E, Montserrat E. Tratamiento con melfalán a altas dosis seguido de rescate con progenitores hematopoyéticos en la amiloidosis primaria [High-dose melphalan treatment followed by hematopoietic progenitor cell rescue in primary amyloidosis]. Med Clin (Barc). 2000 Jul 8;115(6):216-20. Spanish. doi: 10.1016/s0025-7753(00)71514-4.
Excluded (reason: outside the field of interest)

203: Rajani B, Rajani V, Prayson RA. Peripheral nerve amyloidosis in sural nerve biopsies: a clinicopathologic analysis of 13 cases. Arch Pathol Lab Med. 2000 Jan;124(1):114-8. doi: 10.5858/2000-124-0114-PNAISN.
Excluded (reason: outside the field of interest)

204: Saba N, Sutton D, Ross H, Siu S, Crump R, Keating A, Stewart A. High treatment-related mortality in cardiac amyloid patients undergoing autologous stem cell transplant. Bone Marrow Transplant. 1999 Oct;24(8):853-5. doi: 10.1038/sj.bmt.1702005.

Excluded (reason: outside the field of interest)

205: Perfetti V, Colli Vignarelli M, Anesi E, Garini P, Quaglini S, Ascari E, Merlini G. The degrees of plasma cell clonality and marrow infiltration adversely influence the prognosis of AL amyloidosis patients. Haematologica. 1999 Mar;84(3):218-21.
Excluded (reason: outside the field of interest)

206: Kyle RA, Gertz MA, Greipp PR, Witzig TE, Lust JA, Lacy MQ, Therneau TM. Long-term survival (10 years or more) in 30 patients with primary amyloidosis. Blood. 1999 Feb 1;93(3):1062-6.
Excluded (reason: outside the field of interest)

207: Tanaka M, Hongo M, Kinoshita O, Takabayashi Y, Fujii T, Yazaki Y, Isobe M, Sekiguchi M. Iodine-123 metaiodobenzylguanidine scintigraphic assessment of myocardial sympathetic innervation in patients with familial amyloid polyneuropathy. J Am Coll Cardiol. 1997 Jan;29(1):168-74. doi: 10.1016/s0735-1097(96)00438-x.
Excluded (reason: outside the field of interest)

208: Majeed SK. Survey on spontaneous systemic amyloidosis in aging mice. Arzneimittelforschung. 1993 Feb;43(2):170-8.
Excluded (reason: outside the field of interest)

209: Maiorca R, Cancarini GC, Brunori G, Camerini C, Manili L. Morbidity and mortality of CAPD and hemodialysis. Kidney Int Suppl. 1993 Feb;40:S4-15.
Excluded (reason: outside the field of interest)

210: Gertz MA, Kyle RA, Thibodeau SN. Familial amyloidosis: a study of 52 North American-born patients examined during a 30-year period. Mayo Clin Proc. 1992 May;67(5):428-40. doi: 10.1016/s0025-6196(12)60388-7.
Excluded (reason: outside the field of interest)

211: Shimizu K, Komatsu Y. [Overview of hemodialysis: characteristics, indications and recent progress]. Nihon Rinsho. 1991 Dec;49 Suppl:125-32. Japanese.
Excluded (reason: outside the field of interest)

212: Gertz MA, Kyle RA. Hepatic amyloidosis (primary [AL], immunoglobulin light chain): the natural history in 80 patients. Am J Med. 1988 Jul;85(1):73-80. doi: 10.1016/0002-9343(88)90505-0.
Excluded (reason: outside the field of interest)

213: Pellikka PA, Holmes DR Jr, Edwards WD, Nishimura RA, Tajik AJ, Kyle RA. Endomyocardial biopsy in 30 patients with primary amyloidosis and suspected cardiac involvement. Arch Intern Med. 1988 Mar;148(3):662-6.
Excluded (reason: outside the field of interest)

214: Ataman R, Burton PR, Gokal R, Brown CB, Marsh FP, Walls J. Long-term CAPD--some U.K. experience. Clin Nephrol. 1988;30 Suppl 1:S71-5.
Excluded (reason: outside the field of interest)

215: Noël LH, Zingraff J, Bardin T, Atienza C, Kuntz D, Drüeke T. Tissue distribution of dialysis amyloidosis. Clin Nephrol. 1987 Apr;27(4):175-8.
Excluded (reason: outside the field of interest)

216: de Freitas AF. The heart in Portuguese amyloidosis. Postgrad Med J. 1986 Jun;62(728):601-5. doi: 10.1136/pgmj.62.728.601.
Excluded (reason: outside the field of interest)

217: Ruder MA, Alpert MA, Sanfelippo JF, Dix JD, Whiting RB. Symptomatic cardiac amyloidosis in an American family. South Med J. 1984 Jul;77(7):831-3. doi: 10.1097/00007611-198407000-00010.
Excluded (reason: outside the field of interest)

218: Kyle RA, Greipp PR. Amyloidosis (AL). Clinical and laboratory features in 229 cases. Mayo Clin Proc. 1983 Oct;58(10):665-83.
Excluded (reason: outside the field of interest)

219: Majmudar B. Metastasis of cancer to cancer: report of a case. Hum Pathol. 1976 Jan;7(1):117-9. doi: 10.1016/s0046-8177(76)80009-3.
Excluded (reason: outside the field of interest)
